# Exploiting genetic and genomic resources to enhance productivity and abiotic stress adaptation of underutilized pulses

**DOI:** 10.3389/fgene.2023.1193780

**Published:** 2023-06-16

**Authors:** Sangam L. Dwivedi, Mark A. Chapman, Michael T. Abberton, Ufuoma Lydia Akpojotor, Rodomiro Ortiz

**Affiliations:** ^1^ Independent Researcher, Hyderabad, India; ^2^ Biological Sciences, University of Southampton, Southampton, United Kingdom; ^3^ The International Institute of Tropical Agriculture (IITA), Ibadan, Nigeria; ^4^ Department of Plant Breeding, Swedish University of Agricultural Sciences, Alnarp, Sweden

**Keywords:** diversity assessment, gene flow, genes, introgression, nutrition, Orphan legumes, phenomics, quantitative trait loci

## Abstract

Underutilized pulses and their wild relatives are typically stress tolerant and their seeds are packed with protein, fibers, minerals, vitamins, and phytochemicals. The consumption of such nutritionally dense legumes together with cereal-based food may promote global food and nutritional security. However, such species are deficient in a few or several desirable domestication traits thereby reducing their agronomic value, requiring further genetic enhancement for developing productive, nutritionally dense, and climate resilient cultivars. This review article considers 13 underutilized pulses and focuses on their germplasm holdings, diversity, crop-wild-crop gene flow, genome sequencing, syntenic relationships, the potential for breeding and transgenic manipulation, and the genetics of agronomic and stress tolerance traits. Recent progress has shown the potential for crop improvement and food security, for example, the genetic basis of stem determinacy and fragrance in moth bean and rice bean, multiple abiotic stress tolerant traits in horse gram and tepary bean, bruchid resistance in lima bean, low neurotoxin in grass pea, and photoperiod induced flowering and anthocyanin accumulation in adzuki bean have been investigated. Advances in introgression breeding to develop elite genetic stocks of grass pea with low β-ODAP (neurotoxin compound), resistance to *Mungbean yellow mosaic India virus* in black gram using rice bean, and abiotic stress adaptation in common bean, using genes from tepary bean have been carried out. This highlights their potential in wider breeding programs to introduce such traits in locally adapted cultivars. The potential of de-domestication or feralization in the evolution of new variants in these crops are also highlighted.

## 1 Underutilized pulses the key to diversified and climate resilient food system

The world’s food system is threatened by over-dependence on a limited number of crops with low nutritional value and the negative impact of climate change on agriculture (Mayes et al., 2012; Raza et al., 2019). According to World Health Organization (WHO), about 9.9% of the world’s population in 2020 were undernourished this is an increment from 2019s 8.4% and this number has been predicted to increase by 2030 (WHO, 2021). A contributing factor to this problem is the production and consumption of few major crops that results in unbalanced diets lacking enough minerals and nutrients (Temba et al., 2016). Likewise, the edible yield of major crops such as maize, wheat, rice, and potatoes is expected to reduce due to the effects of climate change, which means less food (Thornton and Cramer, 2012; Adhikari et al., 2015). A diversified and climate-resilient food system will enhance sustainable and stable crop productivity while widening the range of nutritious foods (Dawson et al., 2019). This involves but is not limited to the production and consumption of nutritional-rich leguminous crops to complement cereal-based diets, polyculture to enhance soil health, and cultivation of crops that can withstand harsh climatic conditions while producing sufficient yield. All of which can be achieved through the incorporation of underutilized legumes in the food system.

Underutilized legumes—diverse indigenous legumes with good potential that have naturally survived harsh climatic conditions over the years without proper mindfulness—can play an important role in diversifying and improving climate resilience food system (Abberton et al., 2022). They may provide nutritional security due to the high quality of nutritional content they possess (Table 1). They also contain comparable and sometimes superior quantities of essential amino acids, minerals, protein, vitamins, dietary fibers, and some beneficial bioactive substances compared to main legumes (Bhadkaria et al., 2021). Multipurpose underutilized legumes like African yam bean (*Sphenostylis stenocarpa*) and winged bean (*Psophocarpus tetragonolobus*) which produces edible pods, seeds and tubers, and others like yard long bean (*Vigna unguiculata* subsp. *sesquipedalis*), lablab bean (*Lablab purpureus*) and horse gram (*Macrotyloma uniflorum*) which produce seeds and pods can help to diversify the food system (Mustafa et al., 2021). As peculiar to all leguminous crops, they form a relationship through specialized root nodules with symbiotic bacteria, or rhizobia which traps atmospheric nitrogen and converts it to nitrogen which is readily available to the plant (Zhong et al., 2023). Hence, most of them have the ability to grow in poor soils and this has favoured their cultivation over the years (Sandal et al., 2002). The nitrogen remains in the soil after they have been harvested thereby improving the health of the soil for the next planting season (IAEA, 2016).

**TABLE 1:** Nutritional profile of mature raw seeds of underutilized (13) vs. major (5) pulses.

Common name	Carbohydrate (%)	Crude protein (%)	Fat (%)	Energy (Kcal/100 g)	Crude fiber (%)	Ca (mg)	P (mg)	Fe (mg)	Sources
Minor Legumes									
African yam bean	49.9–63.5	19.5–29.5	1.4–7.5	382.4–430.2	2.5–9.6	48.3–85.0	108–130	6.1–10.4	Baiyeri et al. (2018), George et al. (2020)
Adzuki bean	28.5–60.7	16.3–29.2	0.3–1.3	329	12.7	66	381	5	Wang et al. (2022)
Bambara groundnut	53–69	17–25	6.5–8.5	1,609	5–12	30–128	81–563	2–9	Maphosa et al. (2022)
Grass pea	48–52	18–34	0.7–2.8	362	3.9–6.0	220–370	350–640	6.9–8.7	Yan et al. (2006), Grela et al. (2010), Lambein et al. (2019), Ramya et al. (2022)
Horse Gram	50–60	18.5–31.2	0.6–2.6	321	4.3–25.0	244–312	311–443	5.9–7.4	Bhartiya et al. (2015)
Kersting’s groundnut	57.8–81.0	12.9–22.9	1.0–5.3	348	2.0–10.9	103–183	345–392	15	Kafoutchoni et al. (2021), Coulibaly et al. (2022b)
Lablab bean	29.6–66.3	20.535.5	0.3–9.7	344–383	3.7–14	94–132	317–428	1.7–9.4	Davari et al., 2018
									Minde et al. (2021)
Lima bean	49.4–77.34	8.6–30.3	0.5–5.9	338	2–16	68.7–81.0	4.3–11	91.6–128	Gemede and Birhanu (2020), Adebo (2023)
Moth bean	61.5–66.0	22.0–26.0	1.6	343	-	1,144–150	231–489	10.8–15.1	Opara et al. (2017), Bhadkaria et al. (2022)
Rice bean	50–70	14–26	0.5–2.3	347	3.6–5.6	111–598	124–568	3.7–9.2	Dhillon and Tanwar (2018), Pattanayak et al. (2019)
Tepary bean	65–69	21–25	0.9–1.2	360	2.1–3.1	28	450	7.1–8.3	Porch et al. (2017)
Yard long bean	62–83	27–32	1.0–1.8	372–458	1.2–1.8	72–138	59–559	10.3–12.0	Savithiri et al. (2018), Khatun et al. (2022)
Winged bean	12.7–42.2	27–43	13.9–26.7	409	3.4–27.0	102–850	310–637	4.9–6.0	Bepary et al. (2023)
Major Legumes									
Cowpea	50–60	23–32	5.4–11.2	336	3.9–10.6	85–93	438–498	10–11	Jayathilake et al. (2018), Enyiukwu et al. (2020)
Soybean	30.2	36.5–44.4	31.0–71.00	446–470	9.3	277–300	695–704	15.7–16.4	Sharma et al. (2014), Etiosa et al. (2018)
Chickpea	41.1–47.4	19.0–25.0	4.5–6.0	378	18.0–22.0	93–197	263–370	4.6–6.7	Thavarajah and Thavarajah (2012), Jukanti et al. (2012)
Common bean	55.0–65.0	18.5–29.7	0.3–1.3	337	8.5–15.0	75–83	300–750	4.1–10.0	De Almeida Costa et al. (2006), Gouveia et al. (2014), Celmeli et al. (2018)
Lentil	53.0–70.0	24.0–32.0	0.6–3.9	352	1.4–5.9	82–102	281–394	5.9–7.0	Karaköy et al. (2012), Sharma et al. (2022)

Figures rounded up to one decimal point.

Various underutilized legumes are superiorly adapted to marginal areas with high-risk soil and climatic conditions, especially in semi-arid and arid regions of the world, compared to major crops. Therefore, they are tolerant to abiotic stresses such as drought, extreme heat, and poor soils condition, and can grow in areas where the cultivation of major crops is difficult (Aditya et al., 2019). The possession of traits that are responsible for adaptive mechanisms (resistance and tolerance) can be explored for crop improvement. However, a major problem of underutilized legumes is low and inconsistent yield (Cullis and Kunert, 2017). A faster and efficient method to improve the yield of these crop is through employing molecular breeding techniques (Singh et al., 2012). There is a need, therefore, to explore the genomes of these crops to improve their productivity without tampering with their ability to tolerate abiotic stresses. The relatively small genome size of many underutilized crops and recent genomic sequence information of underutilized crops have made exploring the genome of these crops easier. For example, through the information on the genome sequence of some underutilized crops, the genomic mechanism and the number of genes responsible for many agronomic and adaptive genes in underutilized crops have been revealed (Chapman et al., 2022).

Through mixed cropping—which is an option to improve food security as the negative impact of climate change rises—underutilized legumes intercropped with other food crop facilitates higher resource use efficiency to ensure better production and consumption of highly nutritious food crops and promote climatic, pest, and disease resilience. As an example, intercropping maize landraces and Bambara groundnut with the appropriate management practices reduced land and water demand, increased yield and farmers income (Alhassan and Egbe, 2014; Chimonyo et al., 2020). Therefore, together with major crops, underutilized legumes, can help to improve the food system through diversification and production of climate-resilient crops (Talabi et al., 2022).

There is an increased awareness of plant-based diets and substitutes for animal-based protein with plant-based protein. Underutilized legumes possess high protein content and can substitute for animal protein (Sridhar et al., 2022). They can also contribute to healthy living because of their medicinal value. Lablab bean, for example might serve as a suitable dietary option for type II diabetes management (Purwanti et al., 2021) while horse gram contains phytochemicals that can help in the management of hypercholesterolemia, and obesity (Kumar et al., 2013; Malarvizhi et al., 2021). Moth bean (*Vigna aconitifolia*) seeds contain vicilin which is remarkable against pathogenic microorganisms (Ateeq et al., 2022). Grass pea (*Lathyrus sativus*) seeds have been found to prevent cardiovascular disease and cancer tumour development (Lambein et al., 2019). Adzuki bean (*Vigna angularis*) is known as the “weight loss bean” in Asia because it contains low caloric and fat content and can be recommended to people looking to lose weight (Zhao et al., 2021). In combination with cereals, underutilized legumes can help to prevent and manage some health-related issues such as diabetes (Venn and Mann, 2004).

The inclusion of underutilized legumes in our food system through the promotion, cultivation, and consumption can aid to doubling food production by 2050 in order to provide nutritious food to the growing world population. Underutilized legumes are also a promising resource for building a diversified and climate-resilient food system. They have proven to exceed some major crops in yield and nutritional value even while being grown in marginal areas. It is certain that with little research actions, these crops will make their mark in the future of sustainable and resilient agriculture.

This review article includes 13 underutilized pulse species of seven genera (Table 1) and highlights their role in climate resilience and food and nutritional security. It provides up to date information regarding the cataloguing of their genetic resources, assessing their population structure and diversity, as well as it shares knowledge on the advances in throughput phenomics and genomics, identifying major quantitative trait loci (QTL), and getting insights on putative and functionally characterized genes. These advances may accelerate the productivity of these crops without compromising their nutritional and climate resilience characteristics to promote food and nutritional security and livelihoods of those dependent on such crops in marginal lands globally especially in semi-arid and arid regions.

## 2 Domestication, de-domestication or feralization, and re-domestication

The “domestication syndrome” eased the harvest, led to improved nutritional value and facilitated husbandry of today’s major legume crops (Zeder, 2015). Indeed, domestication and further crop evolution influenced edible organs’ size and weight in grain and forage legumes. Humans gave priority also to nutritional and cultural characteristics, thus putting selective pressures on beneficial alleles in each legume crop population. DNA markers such as SSRs and SNPs have been useful to understand broadly the origin and provide insights into the evolution of some species (Zeder, 2006), including those of legumes that provide grains, vegetables and fodder. The diversity in these legume species comes from both natural and artificial selection over time. Population genomics, pan-genomics, gene editing and lipid biochemistry are further unravelling domestication history and adaptive events in legume evolution. The genetic architecture insights will determine genomic regions as selection footprints, which are often related to adaptive functions, e.g. flowering, shattering, feralization and the plant-human interactions (demographic history) during crop evolution.

De-domestication or feralization, which challenge the concept of plant domestication and further crop evolution as a death end, remains under-investigated though feral animal and plants have been known since the introduction of agriculture and are now becoming ubiquitous worldwide (Mabry et al., 2021). Feralization refers to domesticated species that escaped crop husbandry and continue growing in the wild (Ellstrand et al., 2010), but should not be seen just as a domestication reversal, and rather it must be understood as affected by various factors including novel selection pressures (Gering et al., 2019). As such it should be regarded as an extension of crop evolution (Wu et al., 2021). De-domestication *per se* provides, therefore, an opportunity to research adaptive evolution in the legume crops that are growing or invading new habitats or changing environments related to global warming. For example, feral alfalfa or lucerne (*Medicago sativa*) populations are widespread at the roadsides in southern Manitoba (Bagavathiannan and Van Acker, 2009). These feral populations show great genetic diversity, thereby indicating lack of bottlenecks or genetic drift (Bagavathiannan et al., 2010). Furthermore, it seems that they are undergoing selection for adaptative characteristics such as winter survival, rhizome production and prostrate growth habit, which favor their endurance in unmanaged habitats.

Research remains scant on the genomic impacts on feralization and local adaptation as well as gene flow between domesticated, feral and wild populations in plants at large and legumes in particular. A selective advantage under stress allows feral plants to successfully germinate their seed, survive, reproduce and establish a self-perpetuating population. Hence, studying this domestication legacy seen in many feral types will give insights on adaptation to varying sites in minor food legumes, e.g. grass pea or vetch (*Vicia* spp.) in the Mediterranean.

## 3 *Ex-situ* conservation of wild and cultigen gene pool and core collection

*Ex situ* plant conservation is a critical aspect of preserving biodiversity, including but not limited to underutilized crops and crop wild relatives. Whilst *in situ* conservation allows plants to grow in their natural environments, there is always the risk that natural disasters, habitat degradation or changes in human preferences will result in germplasm becoming threatened or extinct (Maxted, 2013). Relevant to this review article, human preferences for modern crops such as wheat and maize may result in abandonment of other native species, including underutilized or minor legumes, as food sources, and potentially the loss of cultivars that are locally adapted (Azam-Ali, 2010).

To preserve species and cultivars, seedbanks and other *ex situ* sites can maintain germplasm, most often as seed, ideally under conditions that maximize the longevity, accompanied by periodic regeneration. In addition to seedbanks, other examples of *ex situ* conservation include orchards and botanic gardens, which typically conserve cultivars and biodiversity in general, respectively.

Based on the publicly available Genesys database of germplasm conserved in genebanks worldwide (https://www.genesys-pgr.org/; accessed February 2023) there are 4.2 million plant accessions conserved. Encouragingly a relatively large number of accessions are not improved cultivars and instead are landraces (20.1%) or wild accessions (10.8%), suggesting that genetic diversity is, at least for some crops and their wild relatives, likely being preserved.

In terms of crops being conserved, the most common species in the genebanks are wheat (*Triticum aestivum*), rice (*Oryza sativa*) and barley (*Hordeum vulgare*), which together comprise nearly 25% of the 4.2 million accessions. In contrast, for the focal minor grain legumes, only tens [Kersting’s groundnut (*Macrotyloma geocarpum*)] to a few thousand [Lima bean (*Phaseolus lunatus*) and grass pea] accessions are conserved (in total 30,623 accessions of these 13 species). Based on a minimum of 10 accessions in a genebank, these 13 crops are found in between one (Kersting’s groundnut) and 29 genebanks (grass pea) (median = 10). The top five genebanks for conserving these minor legumes are the World Vegetable Center (Taiwan, 4551 accessions), Centro Internacional de Agricultura Tropical (Colombia, 3845), Western Regional Plant Introduction Station, USDA-ARS (USA, 3049), International Institute of Tropical Agriculture (Nigeria, 2906), and International Centre for Agricultural Research in Dry Areas (Lebanon, 2557), although for the latter all accessions are grass pea. Overall, these data highlight the risk to some of these crops of both low numbers of accessions being conserved as well as a lack of broad representation in genebanks (Table2). Note also that small regional genebanks and universities and research stations might withhold local accessions of some of these crops, but they are not listed in the Genesys database.

**TABLE 2:** Representation of 13 minor legumes in genebanks (only the top ten for these minor legumes are shown). Data taken from Genesys-PGR (https://www.genesys-pgr.org/; accessed February 2023).

Genebank code	*Sst*	*Van*	*Vsu*	*Lsa*	*Mun*	*Mge*	*Lpu*	*Plu*	*Vac*	*Vum*	*Pac*	*Pte*	*Vuns*	Total
TWN001		2387			54		559	256	26	347	18	284	620	4551
COL003							142	3308		40	355			3845
USA022				294				2275			480			3049
NGA039	495		2032		19	23	64	56		24		193		2906
LBN002				2557										2557
AUS165		350	48	897				153	36	60	107	124		1775
BRA003		29						1284		39	80	15		1447
RUS001		187	16	834				57	64	40	56		135	1389
USA016	12	299	106		33		136		58	43		174	194	1055
UKR008				837					35		17			889
OTHER	97	177	942	2591	87	2	1152	764	133	426	370	47	372	
TOTAL	604	3429	3144	8010	193	25	2053	8153	352	1019	1483	837	1321	

^a^ Gene banks IDs: TWN001 World Vegetable Center, Taiwan), COL003 (Centro Internacional de Agricultura Tropical, Colombia), USA022 (Western Regional Plant Introduction Station, USDA-ARS, United States), NGA039 (International Institute of Tropical Agriculture, Nigeria), LBN002 (International Centre for Agricultural Research in Dry Areas, Lebanon), AUS165 (Australian Grains Genebank, Agriculture Victoria, Australia), BRA003 (Embrapa Recursos Genéticos e Biotecnologia, Brazil), RUS001 (N.I. Vavilov Research Institute of Plant Industry, Russia), USA016 (Plant Genetic Resources Conservation Unit, Southern Regional Plant Introduction Station, University of Georgia, USDA-ARS, United States), UKR008 (Ustymivka Experimental Station of Plant Production, Ukraine).

^b^ Species abbreviations: *Sst, Sphenostylis stenocarpa*; *Van*, *Vigna angularis*; *Vsu*, *Vigna subterranean; Lsa*, *Lathyrus sativus*; *Mun*, *Macrotyloma uniflorum*; *Mge*, *Macrotyloma geocarpum*; *Lpu*, *Lablab purpureus*; *Plu*, *Phaseolus lunatus*; *Vac*, *V. aconitifolia*; *Vum*, *V. umbellate*; *Pac*, *Phaseolus acutifolius*; *Pte*, *Psophocarpus tetragonolobus*; *Vuns*, *V. unguiculata* ssp. *unguiculata* cv.-gr. *sesquipedalis*.

Whilst these *ex situ* conservation approaches conserve germplasm through cold storage and periodic regeneration and checking, and make it available to researchers, there is a risk that if regeneration is poor, the diversity of an accession can become skewed (i.e., the individuals that grow are those that are adapted to the preservation process), or simply reduced, for example if seed from a wild population are grown up and only a tiny number grow to form the next batch of seed. Another arguably more significant risk is that *ex situ* preservation is unlikely to preserve any indigenous knowledge associated with that crop. Therefore, even if the seed are viable, researchers and farmers lack knowledge pertaining to the timing of planting, companion crops, and crop husbandry (Chivenge et al., 2015).

Clonally propagated crops and those recalcitrant to drying and −20°C storage are not present in seedbanks and therefore maintenance of biodiversity for these requires *in situ* conservation, or the setup of dedicated orchards and arboreta. Nevertheless, a recent analysis suggested that the conservation of clonally propagated crop landraces (e.g., yam, sweet potato, banana and yam) was of similar quality (in terms of ecological and geographic variation conserved) as those of cereals and pulses (Ramirez-Villegas et al., 2022), which are commonly preserved in seedbanks.

## 4 Diversity assessment and agronomically beneficial germplasm resource

Plant genetic resources are important sources of variation for crop improvement programs. Understanding the nature of variations, assessing population structure and diversity, and defining genotype × environment interactions (GEI) may lead to identifying genetically diverse, stable, and agronomically beneficial germplasm. Genebanks worldwide contain accessions preserved *ex situ*. Cataloguing variability for such a large pool of genetic resources for morpho-agronomic and physiological traits is very resource-intensive. Moreover, high GEI interaction for many agronomic and seed quality traits requires multiple seasons data to identify germplasm with stable trait expression.

Use of only limited germplasm in crop improvement programs has resulted in a narrow genetic base in many crops. Representative subsets in the form of core or mini core collections (Brown, 1989; Upadhyaya and Ortiz, 2001) have been suggested as a gateway to enhanced utilization of diverse germplasm in crop improvement. Assessing population structure and diversity based on morpho-agronomic and DNA markers combined with appropriate statistical analysis group the germplasm pools into distinct clusters, while DNA markers assessment also provide allelic variation and richness to identify germplasm with specific alleles conferring positive performance (Backiyalakshmi et al., 2021; Sicilia et al., 2022).

### 4.1 Morpho-agronomic traits-based diversity

Various reports detailed assessment of phenotypic diversity (evaluated at least in two environments) following morpho-agronomic descriptors to identify diverse accessions with agronomically beneficial traits in nine underutilized grain legume species (Table 3). Analysis of 169 African yam bean (AYB) accessions involving 31 phenotypic descriptors revealed significant variation for flowering, leaf area, seeds pod^-1^, pod length, seed thickness, and seed weight (Shitta et al., 2021). A detailed morphological characterization of 196 AYB landraces revealed ample genetic variation for flowering, pod and seed size, and seed yield, grouped the accessions into five clusters, and found accessions for earliness, better phenological appeal, and high seed yield. The positive and significant correlation among these traits indicates the possibility of simultaneous improvement (Olomitutu et al., 2022a). Characterization of 40 AYB accessions using 48 descriptors revealed a sizeable proportion of AYB accessions (42%) that produced tubers and four seed shapes were detected. Sixteen significant reproductive traits grouped the accessions into five distinct clusters, and found pods plant^-1^, and total seed weight plant^-1^ as yield determining factors (Ojuederie et al., 2015).

**TABLE 3:** Details of germplasm, environment, morphological descriptor, and significant output reported from phenotypic diversity assessment (at least two seasons’ data) in eight underutilized pulses.

Germplasm (#)	Environment (#)	Descriptor (#)	Output	References
African yam bean (*Sphenostylis stenocarpa*)				
196	6	14	Accessions differing in grain filling period, pod/seed size, pods/seeds plant−^1^, locules pod−^1^, and seed yield identified	Olomitutu et al. (2022b)
169	2	26	TSs62B: highest pods (17.65) plant−^1^ and 100-seeed weight (25.3 g); 3A: earliest to flower (84.50 d)	Shitta et al. (2021)
Adzuki bean (*Vigna angularis*)				
474 (elite, landraces, wild)	2	10	Greater variation for plant growth characteristics in wild adzuki bean as compared to landraces and cultivars; wild adzuki beans were lower yielder than landraces and cultivars; wide variation in flowering, pod/seed size and weight, seed yield	Hu et al. (2022)
61 (wild)	Greenhouse	6 (root traits)	Large differences in root traits (total root length, 82.4–1,435 cm; root surface area, 12.3–208.4 cm^2^; average root diameter 0.23–0.56 mm)	Tayade et al. (2022a)
22 (cultivated)	Greenhouse	6	Significant variation with most traits showing normal distribution; total root length (TRL) showed strong positive correlation with root surface area (RSA) and number of tips (NT); TRL negatively correlated with link average length (LAL)	Tripathi and Kim (2022)
Bambara groundnut (*Vigna subterranea*)				
300 (landraces)	2	16	Landraces could be further improved due to presence of high coefficient of genotypic variation and high heritability for most yield traits; pod and seed traits positively correlated	Uba et al. (2023)
15	3	34	TVSU-455, identified as the best performing accessions (pods plant^-1^ 43; fresh seed weight 46 g plant^-1^, seeds plant^-1^ 47, 100 seed (fresh) weight 124 g, 100 seed (dry) weight 27 g, fresh pod weight 92 plant^-1^ 65, harvest index 0.57, yield plot^-1^ 45.83, unshelled yield plot^-1^ 550 g) across environments	Esan et al. (2023)
100	2 (two water regimes)		Two major clusters and five distinct sub-clusters; geometric mean productivity (GMP) and stress tolerance index (STI) used as measure of drought tolerance	Odesola et al. (2023)
8 (landraces)	2 (rainout shelter; two water regimes)	2 (root traits)	Significant variation in total root length (TRL) and root length density (RLD) under drought stress (DS); RLD under DS substantially increased seed yield, while increase in TRL enable plants to quickly explore water at a deeper soil depth in response to declining soil water	Mateva et al. (2022)
95	2	18	Plant height, leaf length and width, chlorophyll content, pod and seed characteristics contributed most to phenotypic diversity; Southern Africa accessions, great potential to use in breeding	Olanrewaju et al. (2021)
Grass pea (*Lathyrus sativus*)				
94	2	22	Accessions with specific adaptation, low or highland region, with highest biological yield from highland region in Turkey	Arslan et al. (2022)
Kersting’s groundnut (*Macrotyloma geocarpum*)				
297	2	19	Best performing accessions clustered together; pod/seed traits contributed most to variability; moderate to strong positive genetic correlations (0.60–0.96) among pods plant^-1^, seeds plant^-1^, seeds pod^-1^, 100-seed weight, and seed yield	Akohoue et al., 2019
Lablab (*Lablab purpureus*)				
277	2	38	Sufficient variability for pod/seed characteristics (fresh seed colour, green, purple; dry seed shape, round, oval; dry seed colour, 13 types) including fragrance; Tanzania accessions genetically more heterogeneous	Letting et al. (2022)
Rice bean (*V. umbellata*)				
65	2	20	Accessions bordering Myanmar and East Nepal showed wide variability for plant type	Pattanayak et al. (2018)
Winged bean (*Psophocarpus tetragonolobus*)				
4	3	11	An early maturing (68–82 d compared to119-167 d for other), day neutral accession (MYO-01) adapted to Australia environment (October planting, ∼3 t seed yield); seed being hard seededness, a limitation	Eagleton (2022)

Reported only those publications containing at least two seasons’ data.

The genus *Vigna* contains many underutilized pulses (adzuki bean, Bambara groundnut, moth bean, rice bean and others) in addition to some major grain legumes (cowpea and mungbean). Adzuki bean in widely grown across China in a wide range of agroecological environments. A comparison of 475 adzuki bean germplasms including cultigens, landraces and wild relatives revealed significant variation among accessions of different germplasm types, and grouped the accessions into five clusters (Hu et al., 2022). Landraces from the lower Yellow River basin of mid-north China provinces had the greatest diversity, with various levels of cohesiveness (based on phenology, yield and yield components, and plant height data). South China (Sichuan-Anhui) accessions were typically late maturity with low seed weight, while those from north China (Liaoning-Heilongjiang) were early and had a short habit (Redden et al., 2009). Further multisite evaluation of Chinese adzuki bean germplasm showed that late maturing gene pool had the greatest yield at the lower latitude location (Hermitage, Queensland, Australia), while the central Chinese gene pool combined both high yield and acceptable seed quality, thus being suitable for marketing in Japan (Redden et al., 2012). Wild adzuki bean accessions are an excellent source of variation for root system architecture and morphological diversification to enhance the productivity of cultivated adzuki beans. High throughput root imaging analysis of 61 wild adzuki bean accessions showed wide variation (up to 17-fold among contrasting accessions) for root morphological and root architectural traits. Total root length varied from 82 to 1,435 cm, surface area from 12.30 to 208.39 cm^2^, and average diameter from 0.23 to 0.56 mm, whereas root architectural traits, number of tips plant^-1^, link average length (cm), and link average diameter (mm) were 04.33–2549.20, 0.06–0.29, and 0.27–0.61, respectively (Tayade et al., 2022a). Highly significant differences for morpho-agronomic traits among Bambara groundnut landraces and positive correlation among yield and yield attributing traits suggest that landraces could be further improved for agronomic traits (Khan et al., 2021a; Uba et al., 2023).

Hierarchical cluster analysis using 19 morphological descriptors grouped 297 Kersting’s groundnuts from across diverse ecological zones in Benin and Togo, into four clusters (Akohoue et al., 2019). Coulibaly et al. (2022a) integrated ecological niche modeling (ENM) and genetic information to understand the current and future distributions of Kersting’s groundnut populations. Both climatic and soil variables influenced the distribution of Kersting’s groundnut. #Niches projections show divergence in the response of the species and subpopulations to ongoing climate change. Thus, inclusion of genetic information into ENM may help understand species future distribution and adaptation for identifying priority regions for conservation and breeding.

In lablab, wide variation was reported among 277 accessions for pod curvature (from curved to straight), pubescence (glabrous to pubescent), fragrance (absent to high), constriction, colour, attachment, and pod colour at physiological maturity. Diverse seed colours and shapes were noted. Cluster analysis based on 14 quantitative traits grouped the accessions into four groups, with weak association between group membership and the place of origin. Variation between clusters meant that, for example, one cluster had more locules pod^-1^ and seeds pod^-1^, and another had a greater number of pods and yield (Letting et al., 2022). Gene pools with 2 seeds per pod and 4 or more seeds per pod are the result of independent domestication events (Njaci et al., 2023).

Arslan et al. (2022) noted significant variability for quantitative and qualitative traits among 94 grass pea accessions evaluated at low- and high-land Turkey environments. Accessions with low β-N-oxalyl-L-α, β-diaminopropionic acid (β-ODAP) content, a neurotoxin compound, were late in flowering and produced low biomass and seed yields. Resistance to broomrape (*Orobanche* spp.) was investigated in grass peas because this weed is a root holoparasitic plant that cause substantial loss to pulses production in the Mediterranean and sub-Saharan Africa. Assessment of wild grass pea accessions against two common broomrape species (*O. foetida*, *O. crenata*) detected complete resistance to *O. crenata* and *O. foetida* in *Lathyrus articulatus* and moderate resistance in *L. aphaca* and *L. ochrus* (Abdallah et al., 2021).

Multivariate analysis based on phenotypic descriptors grouped 48 Brazilian lima bean landraces into three clusters. UFPI-667 and UFPI-682 are genetically distinct and complementary in their characteristics, shorter cycle or high yield, which may be intercrossed to developed new populations (Assunçäo Filho et al., 2022). Thirty-one lima bean germplasm showed substantial variation in 100-seed weight (24–72 g) and protein content (20%–30%). Tannin content varied tenfold among the accessions that increased with seed color (Offei et al., 2006). A few lima bean landraces from the Yucatan peninsula (Mexico) when evaluated for leaf and physiological traits, herbivory insect damage and seed yield, were noticed as the best performing landraces, combining lowest cumulative herbivorous damage and high seed yield. Morphological (leaf number, area, dry mass of leaves; trichome density, specific leaf thickness, hardness) and physiological (photosynthesis rate, stomatal conductance, intercellular carbon, water use efficiency, transpiration) characteristics positively correlated with low insect damage and high seed yield (Ruiz-Santiago et al., 2021).

Tepary bean accessions (six cultivated and 19 wild among 302 accessions evaluated) grown under high temperature and acid soil conditions with aluminum toxicity produced more pods plant^-1^, larger seeds, and a greater number of seeds pod^-1^. These accessions revealed significant differences in physiological traits, such as flowering and maturity, specific leaf area, stomatal density, and root biomass. In addition, cultivated accessions had higher photochemical quenching (qP), while energy dissipation by non-photochemical quenching (NPQ) in the form of heat and the coefficient of non-photochemical dissipation (qN) were higher in cultivated regressive and wild accessions, which probably contributed to differences in adaptation to combined stress of high temperature and acidic soil conditions. Six and 19 accessions of cultivated and wild groups, respectively, had grain yields above 1.8 t ha^-1^, which may be deployed in breeding program to improve productivity of tepary beans in such environments (Suárez et al., 2022).

The daylength-neutral accession MY0-01 from Bago (Myanmar) is adapted to southern Australia and is an excellent genetic resource to breed photoperiod sensitive winged bean cultivars. The small pod and hard-seededness, respectively, may lower its potential for vegetable production and adversely affect germination and plant establishment (Eagleton, 2022).

### 4.2 Molecular-based diversity

Table 4 lists DNA marker-based assessment of population structure and diversity among select germplasm to identify genetically diverse accessions in 13 underutilized grain legume species.

**TABLE 4:** Details of germplasm, DNA markers, and significant output reported from molecular-based diversity assessment in underutilized pulses.

Germplasm no.	Markers no.	Output	References
African yam bean (*Sphenostylis stenocarpa*)			
169	1789 SNPs^a^	Sub-populations with significant genetic differentiation; high similarity between phenotype and genotype-based matrices	Shitta et al. (2022)
93	3722 SNPs	Highly predictive genotypic-phenotypic diversity relationships	Aina et al. (2021)
77	AFLPs^b^	59 of 227 AFLP bands polymorphic; genetic distance between 0.048 and 0.842; four distinct clusters, not related to their geographical origin	Adewale et al. (2015)
Adzuki bean (*Vigna angularis*)			
261	110 SSR^c^	North and South China accessions sufficiently differentiated	Chen et al. (2015)
96	26 SSR	Sufficient similarity between Chinese and Japanese wild relatives	Liu et al. (2014)
176	85 SSR	Higher allelic diversity in the wild than cultivated germplasm	Wang et al. (2012)
Bambara groundnut (*Vigna subterranea*)			
100	5,927 DArTseq SNPs	Two distinct clusters, with cluster I contain TVSu-1897, the remaining in cluster II based on DArTseq SNPs and stress tolerance index	Odesola et al. (2023)
100	5925 SNPs	Higher genetic diversity among Nigerian accessions	Osundare et al. (2022)
93	2286 SNPs + morpho-agronomic traits	Two heterotic groups and unique accessions with specific characteristics	Majola et al. (2022)
270	3,343. DArT^d^ SNPs	Greater diversity among accessions from diverse regions	Uba et al. (2021)
78	19 SSR	Higher within landrace diversity than between landraces; significant gene flow among the landraces	Minnaar-Ontong et al. (2021)
96	32 ISSR^e^	Moderate to high levels of genetic differentiation	Khan et al. (2021b)
*Grass pea* (*Lathyrus sativus*)			
400	56 SSR	Highly polymorphic and diverse germplasm	Rahman et al. (2022)
22	31 SSR	A few genetically diverse germplasms with low β-ODAP identified	Arslan et al. (2020)
118	18 EST-SSR^f^	Sufficiently differentiated high- and low-β-ODAP (neurotoxin) accessions	Gupta et al. (2018)
283	30 SSR	Wild relatives clustered separately from cultigens	Wang et al. (2015)
Horse Gram (Macrotyloma uniflorum)			
58	150 SSR	Three to four distinct genetic stocks	Kumar et al. (2020a)
48	117 SSR	Within population genetic variance greater than between populations	Kaldate et al. (2017)
360	33 SSR & 24 morphological descriptors	Two distinct gene pools with higher levels among accessions variability	Chahota et al. (2017)
Kersting’s groundnut (*Macrotyloma geocarpum*)			
217	886 DArTseq	High level of differentiation among populations and morphotypes; eight distinct clusters	Kafoutchoni et al. (2021)
281	493 SNPs	Distinct subpopulation structure differentiated by seed coat colour and/or ecological regions	Akohoue et al., 2020
Lablab (*Lablab purpureus*)			
166	2460 SNPs	Four distinct subpopulations differentiated by ecogeographic regions	Njaci et al. (2023)
142	1,000 SNPs	Substantial among accessions diversity than within accession variance	Muktar et al. (2021)
65	9320 DArTseq-based SNPs & 15,719 SilicoDart markers	Lower discrimination; higher within population variance than among population	Sserumaga et al. (2021)
Lima bean (*Phaseolus lunatus*)			
183 landraces	12 SSR & 7 morphological descriptors	Sufficient discrimination and introgression between Andean and Mesoamerican genepools	Silva et al. (2019)
46	73 ISSR	Higher genetic diversity in Mayan lowland than Mayan highland landraces; Mayan culture shaped diversity	Camacho-Pérez et al. (2018)
67 wild populations	10 SSR	Higher among populations than within population variance; geographically divergent populations	Martínez-Castillo et al. (2014)
11 wild populations	8 SSR	Strong population differentiation due to geographical isolation	Martínez-Castillo et al. (2006)
Rice bean (*Vigna umbellata*)			
65	28 SSR	High diversity among North-Eastern hill accessions with exceptional outcrossing	Iangrai et al. (2017)
388 (cultigen)	13 SSR	Greater diversity in wild than cultigen populations	Tian et al. (2013)
84 (wild)			
Tepary bean (*Phaseolus acutifolius*)			
20	10 SSR	Moderate differentiation; SSR- and morpho-based-diversity assessment well correlated	Mhlaba et al. (2018)
158 (tepary)	768-SNP	Greater insight into the structure and its relationship with common bean	Gujaria-Verma et al. (2016)
6 (common bean)			
Winged bean (*P. tetragonolobus*)			
457	14 SSR	Moderate gene diversity with high genetic admixture; subpopulations unrelated to geographical origin	Laosatit et al. (2022)
124	13 genic SSR	Comparable gene diversity between Thailand and exotic winged bean accessions	Sriwichai et al. (2021)
Yard long bean (*V. unguiculata* ssp. *unguiculata* cv.-*gr. Sesquipedalis*)			
84	26 InDels^g^	Differentiated salt tolerant and sensitive accessions	Zhang et al. (2021)

^a^ Single nucleotide polymorphisms.

^b^ Amplified fragment length polymorphisms.

^c^ Simple sequence repeats.

^d^ Diversity arrays technology.

^e^ Inter simple sequence repeats.

^f^ Expressed sequence tags.

^g^ Insertion Deletion.

Single nucleotide polymorphism (SNP)-based diversity assessment involving 169 AYB grouped the accessions into three subpopulations with high genetic differentiation. Subpopulation 1 accessions were high yielding, while those in those in subpopulation 2 were highly polymorphic and heterozygous (Shitta et al., 2022). Another study involving 93 AYB accessions and 3722 SNPs and multiple clustering methods detected substantial genetic diversity and formed three to four clusters, with most accessions in each cluster having a similar phenotype, i.e., seed or seed and tuber types (Aina et al., 2021). Insertion-deletion (InDel) markers differentiated salt tolerant from sensitive AYB accessions (Zhang et al., 2021).

A study involving 261 adzuki bean accessions from China and 163 simple sequence repeats (SSRs) grouped the accessions into 10 clusters. Accessions from northern China were genetically distinct than those from southern China (Chen et al., 2015). Wild adzuki beans had higher allelic diversity than cultivated types. Structure analysis clearly separated the wild from the cultigen germplasm, with subdivisions in cultigens based on ecological regions of adaptation (Wang et al., 2012). High genetic differentiation was reported between wild adzuki beans and their wild relative *Vigna minima*. Accessions could be distinguished from each other based on their origins, which suggests that geographic regions of adaptation of wild adzuki bean shaped their genetic variation. Further unfolding of relationship between Chinese adzuki bean cultivars and wild adzuki bean accessions highlights their closeness to Japanese wild adzuki beans than to domestic accessions, suggesting greater involvement of Japanese adzuki bean accessions in Chinese adzuki bean breeding (Liu et al., 2014).

Profiling of 93 South African Bambara groundnut accessions using 2,286 SNPs and morpho-agronomic traits, revealed moderate genetic differentiation and two distinct clusters (Majola et al., 2022). Highly polymorphic SNPs (5927) differentiated 100 Nigerian Bambara groundnut accessions into seven subpopulations and significant marker-trait associations (MTAs) for several morpho-agronomic traits (Osundare et al., 2022). DArT SNP profiling of 270 Bambara groundnut landraces revealed three subpopulations which corresponded to geography. Accessions from West Africa and of unknown origin formed subpopulation 1, Central Africa accessions subpopulation 2, and those from southern and eastern Africa subpopulation 3 (Uba et al., 2021).

Validation and diversity analysis involving 33 polymorphic SSRs and 58 horse gram germplasm detected wide variation and grouped the accessions into 3 (Structure analysis) or 4 (UPGMA and PCA) distinct clusters. The early flowering types are clearly separated from late flowering group (Kumar R. et al., 2020). Another study involving 48 horse gram germplasm and 117 SSRs grouped the accessions into two distinct groups (Kaldate et al., 2017). High quality SNP (493) data on 281 Kersting’s groundnut accessions formed four clusters (based on a neighbor joining tree), which were differentiated by seed coat colour, while structure analysis, yielded two subpopulations. Most of the accessions from the Sudan savanna were in subpopulation I, while those from Sudano-Guinean and the Guinean savannas were in subpopulation II. This study also detected 10 significant MTAs, of which six SNPs were consistent across environment (Akohoue et al., 2020).

Profiling of 25,039 DNA markers (9320 DArT SNPs and 15,719 SilicoDart) data on 65 lablab bean germplasm showed low discriminating ability and three subpopulations unrelated to site of origin. High within population variance suggests a greater degree of gene exchange or low genetic differentiation among the populations (Sserumaga et al., 2021). Another data set involving 1,000 SNP and SilicoDArT markers data on 142 lablab accessions unfolded five major groups, each with further subgroups (Muktar et al., 2021).

The presence of β-N-oxalyl-L-a,b-diaminopropionic acid (β-ODAP) causes neurological disorders in humans. Analysis of 56 SSRs on 400 accessions of grass pea germplasm showed highly diverse structure, with two main and one admixed population (Rahman et al., 2022). Analysis of 31 EST-SSRs data on 22 grass pea accessions having low β-ODAP content detected two main clusters, with high genetic distance between some pairs of accessions, which may be used in crop improvement program to develop populations with large variation to pursue selection for low β-ODAP content (Arslan et al., 2020). Eighteen EST-SSR data from 118 accessions including wild relatives formed four clusters. High β-ODAP accessions (mostly wild types) were in cluster I and those with low β-ODAP in cluster II (Gupta et al., 2018). A large study using 30 SSRs grouped 283 grass pea accessions into three clusters, i.e., wild species, Asian accessions, and Europe and Africa accessions. Asian accessions were clearly separated from other groups (Wang et al., 2015).

Lima bean germplasm was extensively analyzed for assessing population structure and diversity. A combined analysis of genotyping (12 SSRs) and phenotyping (7 morphological descriptors) data of 183 Brazilian lima bean landraces detected high diversity and three distinct subpopulations, one predominantly from the Andean gene pool with large seeds (mean 100-seed weight 80 g) and the other two predominantly from the Mesoamerican gene pool (mean 100-seed weight 34 g), with considerable introgression between the Andean and the Mesoamerican gene pools (Silva et al., 2019). Analysis of 73 inter simple sequence repeats (ISSRs) on 46 Mayan lima bean landraces detected high levels of diversity and genetic differentiation. Mayan low landraces showed higher genetic diversity than Mayan high landraces, thereby indicating the influence of Mayan culture on diversification and conservation of lima beans (Camacho-Pérez et al., 2018). Wild lima beans have two gene pools, known as MI and MII. Genomic profiling of 67 wild populations of lima bean from Mexico with 10 SSR markers, however, unfolded three gene pools, MI1a, MI1b, and MII, with greater among population than within populations variance. MI and MII were geographically divergent, while MI1a and MI1b overlap in their distribution and the presence of admix individuals suggests geneflow among gene pools (Martínez-Castillo et al., 2014).

Molecular profiling of 472 rice bean accessions (388 cultivated and 84 wild) detected high gene diversity in cultivated populations, which was ∼83% of that for wild populations. East Asian populations formed a distinct gene pool. The cultivars from Indonesia had a genetic structure like the wild accessions. Accessions from western Nepal were quite distinct from others and formed a specific group, thereby being a unique gene source for rice bean breeding (Tian et al., 2013). Northeastern Himalayan hills of India are the hotspot for biological diversity. The rice bean populations from this region showed exceptionally high outcrossing rate. Characterization of 65 rice bean accessions from this region with 28 SSRs revealed high mean gene diversity bordering eastern Nepal and Myanmar, formed three distinct clusters, with accessions bordering Myanmar and eastern Nepal in a distinct cluster (Iangrai et al., 2017).

Molecular profiling of 158 cultivated and wild tepary bean populations with high quality SNPs (768) separated domesticated and cultivated tepary beans, with two distinct groups within the domesticated types (Gujaria-Verma et al., 2016). Furthermore, characterization of 20 cultivated accessions with ten highly polymorphic SSRs revealed moderate differentiation among genotypes. SSR-based diversity correlated with diversity assessment based on morphological descriptors, and a few genetically distinct accessions for use in breeding (Mhlaba et al., 2018).

Molecular profiling of 457 accessions winged bean accessions with 14 SSRs revealed moderate gene diversity and high genetic admixture. Seed exchange and relatively high outcrossing probably contributed to high genetic admixture in this germplasm set. Structure analysis grouped the accessions into three subpopulations, unrelated to geographic origins, with most accessions having long pods (30 cm or greater in length), purple seed coats or young purple pods grouped together (Laosatit et al., 2022).

Overall, assessment of population structure and diversity among gene pools provides greater insights into the genetic makeup of populations, which will help in conservation strategy, management, and utilization of diversity in crops breeding and genetics. Unlike major pulses where there has been extensive use of genetic resources in crop breeding, the diversity in underutilized pulses remains largely untapped as a source of alleles for introgression. Hence the primary gene pool can harbor unexploited genes for crop improvement.

### 4.3 Seed nutritional diversity

The declining nutritional quality and bioavailability of nutrients results in serious malnutrition (Dwivedi et al., 2013; Owino et al., 2022). Underutilized pulse grains are rich sources of seed-protein, minerals, vitamins, and phytonutrients, and vary in hydration, cooking, textural and pasting properties. For example, seed weight and protein content among adzuki bean accessions, respectively, varied from 75 to 148 g 1,000 seeds^-1^ and 19%–24%. The accessions also showed significant variation in their physicochemical, cooking hydration and textural properties. Soaked grain hardness ranged between 69 and 120 N and had significant positive association with gumminess and chewiness (Yadav et al., 2018). Assessment of physicochemical and digestive properties of starches from Japanese “dainagon” adzuki bean variants revealed larger particle size in “Noto-dainagon” and “Kyoto-dainagon” starch types were easily gelatinized, none of their starch showed breakdown, and ‘dainagon’ starch was more indigestible than others (Honda et al., 2020). Adzuki bean seedcoats are rich in phenolics and antioxidant compounds, thus having a potential health benefit to humans (Johnson et al., 2022). Total metabolites, the saponin and phenolics contents, ranged from 16 to 945 mg DE g^-1^ and 0.80–57.35 mg GAE g^-1^, respectively. Delphinidin-3-O-glucoside and delphinidin-3-O-galactoside were the predominant anthocyanins in black-seeded adzuki bean cultivars (Desta et al., 2022). Seed coat is a rich source of metabolites and closely associated with the anthocyanin and flavonoid metabolism pathways. Analysis of 10 seed coat colour types in adzuki bean including red, black mottle or gray, golden, green, black, ivory, brown, and light brown revealed anthocyanins the main pigment source, with no carotenoid or pelargonidin derivatives in the seed coats. The pigment composition of the different seed coat colours and the combination of proanthocyanidins and anthocyanins affect the seed coat colour in adzuki bean (Zhao et al., 2022).

Bambara groundnut landraces showed considerable differences in total phenolics (0.75–17.71 mg GAE g^-1^), flavonoids (0.01–2.51 mg QUE g^-1^) and anthocyanins (0.03–1.31 mg CYE g^-1^), with caffeic and catechin discriminating the landraces (Tsamo et al., 2018), while a comparison of phenolic in whole and dehulled Bambara groundnut cultivars differing in seed colour varied from 3.6 to 11.0 GAE g^-1^ and from 2.7 to 3.2 GAE g^-1^, respectively (Adedayo et al., 2021). A new compound, luteolin (C_20_H_18_O_9_), with antioxidant activity discovered in Bambara groundnut suggests that seed could be used as natural sources of antioxidants to reduce inflammation in humans (Chinnapun and Sakorn, 2022).

The seed protein content of grass pea accessions belonging to both *L. sativus* and *L. cicera* ranged between 25% and 30%. The protein is of high quality, except for having low methionine. The seeds are not rich in fat (<1%) but contain high level of polyunsaturated fatty acids. *L sativus* accessions had relatively low level of β-ODAP content, average 733 mg with a range of 0.583–1.340 mg kg^-1^ DM, while those from *L. cicero* accessions had slightly higher β-ODAP, average 1,168 mg with a range of 0.911–1.349 mg kg^-1^ DM. *L. cicera* seeds on average contain greater (6.4 g kg^-1^ DM) tannins than *L. sativus* seeds, 3.3 g kg^-1^ DM. The tannin content in *L. sativus* seeds were significantly correlated with flowers and seeds colour, darker colour associated with higher levels of tannins (Grela et al., 2012). Assessment of seed protein and β-ODAP contents among 702 *L. sativus* accessions revealed significant differences in seed protein (28.82%–30.72%) and β-ODAP (0.32%–0.47% and 0.38%–0.53%), and reported accessions low in seed protein and β-ODAP contents (Rajendran et al., 2019). The landraces from Turkey showed greater variability in seed protein (24.07–30.9%) and β-ODAP (1.35–3.86 mg g^-1^ DM) contents, with a few identified as low in seed β-ODAP and high protein contents (Basaran et al., 2011).

The protein content among 96 diverse horse gram germplasm ranged from 13% to 40%, with the highest protein noted in the wild species *Macrotyloma sar-gharwalensis*. The number of metabolites varied from 25 to 44, and the most nutritionally diverse germplasm amongst the panel were IC 280031 and IC 139356, with the greatest number of quantifiable metabolites, which may be used for the development of nutraceutical food for humans. Chemo-markers such as methionine, sucrose, maltose, riboflavin, and myricetin effectively differentiated this panel (Gautam and Chahota, 2022). Assessing the chemo-diversity of horse gram germplasm cultivated for eight seasons in the Alpine Himalayas unfolded 46 diverse metabolites, including 18 amino acids, nine carbohydrates, three vitamins, seven flavonoids, and nine miscellaneous molecules, which generated a single snapshot chemical signature to differentiate accessions. Higher elevation (1829 m asl) seed-produce resulted in greater amounts of metabolites (55.78 g kg^-1^ DM), than those obtained from lower (1,000–1,150 and 1,150–1,450 m asl). Chemotype-based PCA formed three distinct clusters, with cluster 3 accessions containing the nutritionally best metabolites (Gautam et al., 2022). Horse gram seed is a good source of bioactive and nutritive compounds compared to other pulses and has a high level of antioxidant and radical scavenging activities with immense health benefits (Ingle et al., 2021).

Variation in fatty acid profiling of lablab bean suggests its grain is the good source of essential omega-6 fatty acid linoleic acid (C18:3, ω-6) and omega-3 linolenic acid (C18:3, ω-3), in addition to containing high amounts (24%) of proteins and minerals but low (1%) in phytate content (Hossain et al., 2016; Kumari M. et al., 2022). Low phytate content in the seed enhances mineral bioavailability, which may mitigate nutritional vulnerability in the developing world (Dwivedi and Ortiz, 2021). Significant variability in phytochemicals: i.e., trypsin inhibitors (0.041–0.078 TIU mg^-1^), phytic acid (0.26–1.22 mg g^-1^ DM), radical scavenging activity (5.63–38.26), and tannins (1.4–3.2 mg g-1 DM), were reported among moth bean germplasm (Gupta et al., 2016).

Bhagyawant et al. (2019) noted significant variation in seed protein (2.88–31.79 mg g^-1^ DM) content and antioxidant activity (9.36–18.56%) among rice bean germplasm, with some accessions showing highest *in vitro* protein (63.84%) and starch (44.6%) digestibility. Accessions with high antioxidant activity, protein content, increased *in vitro* protein and starch digestibility and reduced oligosaccharides could be used as a donor parent or promote them for human consumption.

Assessment of wild and domesticated tepary bean accessions for 100-seed weight and protein contents revealed higher level of protein and lower seed weight in wild than domesticated tepary beans. However, domesticated tepary beans had a larger range variation in protein values than the wild tepary beans (Waines, 1978). Tepary bean relative to common bean showed reduced fat and ash concentration but higher sucrose content. Shorter cooking time and a high percentage of seeds with measurable water uptake were reported in tepary bean than common bean accessions, while a few lines were of ‘hard-shell’ trait (i.e., low water uptake) and longer cooking time. Tepary bean is highly nutritious, with nutrient composition and cooking characteristics like that of common bean (Porch et al., 2017). A few common bean × tepary bean interspecific congruity-backcross lines (#12, 76, 77, 78), though yielded poorly, had seed-Fe content over 100 mg kg^-1^ DW (Burbano-Erazo et al., 2021). Hence greater efforts are needed to form such subsets in other underutilized pulses to enhance germplasm use in crop improvement programs.

### 4.4 Representative subset

Reduced core and mini core subsets (defined by morphological descriptors or genotyping data and representing the diversity of the entire collection of a species accessions preserved in a genebank) are the ideal set of genetic resources for detailed characterization to dissect population structure and diversity, discovering trait-specific accessions, gene discovery and allele mining. The underutilized pulses, unlike major grain legume crops, received low priority in management and utilization of genetic resources in genetic enhancement programs. To date, core collections have been reported in adzuki bean (Ning et al., 2008; Takeya et al., 2013), Bambara groundnut (Uba et al., 2023), lablab bean (Vaijayanthi et al., 2015), lima bean (Gomes et al., 2020), and moth bean (Meghwal et al., 2015).

## 5 Gene flow and wild-crop introgression on structure and diversity

Genetic variation can increase through the formation of new genetic combinations resulting from gene flow or introgression between domesticated species and their wild relatives. These combinations of hybrids that survive through time expand the genetic diversity of domesticated crops, serve as the basis for the evolution of domesticated species, and carry traits that can be exploited by breeders (Heredia-Pech et al., 2022). There must be genetic compatibility between domesticated species and their wild relatives for introgression or gene flow to take place (Félix et al., 2014).

Gene flow between wild relatives and domesticated species occurs in regions where both species are found; this could be centers of diversity especially where traditional cultivation is still practiced (Chacón-Sánchez et al., 2021). The suggested wild relatives of some underutilized legumes, e.g. winged bean, are not found in the same area as the domesticated species thereby strongly reducing the chance of crop-wild gene flow (Yang et al., 2018; Sriwichai et al., 2021). However, the genetic bases of winged bean can be expanded through introgression of novel genes from wild relatives into winged bean to develop resilient and efficient winged bean lines with improved yield (Tsoutsoura et al., 2022). The effect of gene flow and crop-wild introgression on populations and genetic diversity relies on the degree and direction of gene movement and could either be a positive or a negative effect (Heredia-Pech et al., 2022). The higher the degree of gene flow the greater its effect on population structure and diversity.

SSR marker and SNPs have been used to estimate the degree and direction of gene flow in major and minor crops (Hübner et al., 2012; Kumar D. et al., 2020). Research by Heredia-Pech et al. (2022) to find the effect of gene flow and introgression on the genetic structure and diversity of lima bean (*P. lunatus*) using SSRs in both local and regional scales at the Yucatan Peninsula region of Mexico concluded that there was evidence of a bidirectional gene flow at the local scale, as well as an asymmetry in crop-to-wild introgression in some accessions. However, these processes do not seem to be affecting the genetic structure and diversity of the species in the longer term, which could be because of low gene flow. Furthermore, results from Heredia-Pech et al. (2022) suggest that gene flow and introgression can be playing an important role at the local scale.

Strong gene flow and introgression from wild relatives into domesticated species bring about increased genetic diversity which can cause heterogeneity in the population structure of domesticated species. This could also increase the similarity of populations that are geographically distant thereby reducing genetic differentiation between populations and increase the genetic diversity of a larger, more interconnected population (Smith et al., 2020). Additionally, this could enable a wide adaptation of domesticated species across diverse environmental conditions but could be to the disadvantage of the wild relative when it is on the receiving end by causing reduced fitness in these environmental conditions as a result of beneficial gene suppression. This disadvantage could eventually affect species persistence, reduce genetic diversity, cause extinction of wild relatives’ population, and the development of aggressive weedy varieties (Martínez-Castillo et al., 2007; Kremer et al., 2012). However, it can sometimes lead to ecological fitness of the wild population (Chacón-Sánchez et al., 2021). All of these can depend on population size, genetic variation, and the environment (Sexton et al., 2014).

An advantage of gene flow and introgression can be seen in the work of Wang et al. (2004). They studied gene flow and introgression in Adzuki bean using microsatellite markers and concluded that introgression is the cause of higher genetic diversity among the offspring from natural outcrossing between cultivated and wild forms (Wang et al., 2004). In addition, the introgressed populations obtained by crossing tepary bean with common bean were found tolerant to drought and subzero temperatures, with several performing better than common bean parents under both stress conditions (Souter et al., 2017).

Finally, this reveals that gene flow and introgression which formulate new genetic combinations may contribute to increase in the ecological fitness of wild species and adaptation of domesticated species to harsh weather conditions. In addition, gene flow and introgression can be useful in the improvement of underutilized legumes and their wild relative as shown in the introgression between tepary bean and common bean. Evidently, strong gene flow and introgression from wild-crop type can be advantageous in breeding for underutilized legumes cultivars that are high-yielding under abiotic stress. With the advent of molecular breeding, the process of gene flow and introgression could be faster, easier and cheaper, thereby ensuring the fat release of underutilized legume varieties well adapted to biotic and abiotic stress. (Martínez-Castillo et al., 2007).

## 6 Genomic resources including resequencing of gene pool diversity

### 6.1 Genetic markers and maps

The availability of genetic markers discussed above allows evolutionary and genomic insight into crop origins and the genetic basis of adaptive traits. These markers have revolutionised the identification of QTL; i.e., regions of the genome controlling one or more traits of interest (Paterson et al., 1988). Small numbers of genetic markers can give coarse resolution of QTL, but as more become available the genomic interval controlling a particular trait can be narrowed down, sometimes to the causative gene (reviewed in Burke et al., 2007). Research using this approach was the first to identify genes underlying important agronomic traits, e.g. the loss of shattering in domesticated rice (Konishi et al., 2006; Li et al., 2006), hard kernel coat in maize (Wang et al., 2005), and fruit size in tomato (Frary et al., 2000). High density linkage maps are available for several minor legumes, including adzuki bean (Han et al., 2005), Bambara groundnut (Gao et al., 2023), grass pea (Hao et al., 2022), lima bean (Garcia et al., 2021), and winged bean (Chankaew et al., 2022).

### 6.2 Genomes and pangenomes

Even dense linkage maps may give poor resolution of causative genes if recombination is low, or the number of plants required is prohibitively large (the rice examples above both required >10,000 plants, which would be unreasonable for species where the plants are larger). However, identifying a genomic region and knowing the sequence of the entire region can remedy this, allowing the researchers to find candidate genes based on likely gene function. The sequencing, assembly and annotation of crop genomes is therefore a significant step in any analysis of agronomic traits. Once available, this can fast-track the identification of causative genes and alleles, as well as identify markers for marker-aided breeding (Jackson et al., 2011).

Whilst modern high throughput sequencing technologies can sequence a genome many times over for a fraction of the cost required for the first genome assemblies, the most accurate and complete assembly requires significant investment, aided by very long read sequencing, and large mapping populations to help unify scaffolds into chromosomes. This means that while many minor legume crops have been investigated with genome sequencing, the availability of high-quality contiguous genome sequences of minor legumes is low, with the two best examples probably being pigeonpea and chickpea, which, due to their widespread use and research investment, probably cannot be considered ‘minor’ legumes. Therefore, focussing on those in Table 2, we can see that adzuki bean (Kang et al., 2015; Yang et al., 2015), lima bean (Garcia et al., 2021), tepary bean (Moghaddam et al., 2021), horse Gram (Shirasawa et al., 2021), and lablab (Njaci et al., 2023) have genomes available with a good contiguity and coverage, and grass pea has a recent attempt to assemble this very large genome (Rajarammohan et al., 2023). Several more are in progress (see Chapman et al., 2022), for example Bambara groundnut (Salazar-Licea et al., 2022), and winged bean (Tsoutsoura et al., 2022).

For any species it is important to remember that a reference genome, even when contiguity and coverage are very high, only represents one individual, and there are expected to be genomic regions in other individuals that are absent from a single reference. This relates to the concept of pangenomes, wherein a complete species’ genome can only be estimated when a range of accessions are investigated (Della Coletta et al., 2021; Chapman et al., 2022). Genomic regions present in only some accessions are termed ‘dispensable’ and are typically highly repetitive and with a low density of coding regions (Gao et al., 2019). Despite this, they can contain enriched gene ontology categories of important gene types, for example defense-related genes in *Brachypodium* (Gordon et al., 2017) and an important flavour-related gene in tomato (Gao et al., 2019).

Whilst a highly complete and contiguous genome is desirable, the cost and resources required can be prohibitive. A low coverage and fragmented genome can be generated for a fraction of the cost, for example only using Illumina-based short-read sequencing. Despite repetitive regions of the genome prevent the assembly of larger fragments, this fragmented assembly can be gene rich and used to identify thousands of molecular markers and complete coding regions that are useful for investigations into the crop diversity and variability and understanding coding sequence-based selection across species during their evolution (Fisher et al., 2022).

### 6.3 Population genomics and GWAS

Genetic markers are useful for understanding the partitioning of genetic variation among crop accessions as well as identifying closely related wild taxa that may be useful for breeding. Genetic markers, often SSRs and SNPs have been used in a variety of minor legumes to understand domestication, gene flow and population level variation (Table 4).

Carrying out whole genome resequencing of a broad range of germplasm and mapping this back to the reference genome, i.e., population genomics, ensures that much larger numbers of genetic markers, potentially hundreds of thousands to millions, are incorporated into the analysis. Through this, and if the sampling is appropriate, more detailed information on crop origins can be determined, for example in the underutilised crops pearl millet (Varshney et al., 2017), fonio millet (Abrouk et al., 2020) and Guinea yam (Sugihara et al., 2020). Whilst minor legumes are further understudied relative to these, recent work using population genomics has, for example, confirmed two origins of domesticated lablab (Njaci et al., 2023). A further advantage of this depth and breadth of sequencing is the ability to correlated genotype and phenotype in a genome-wide association study (GWAS). This approach has been employed in several crops, including some minor legumes too. Through this the genetic basis of reduced alkaloids in lupin (Wang et al., 2021) and flowering time in rice bean (Guan et al., 2022) have been, in part, resolved.

### 6.4 Syntenic relationships among closely related species within and between genera

Genome evolution can involve indels, inversions, translocations, and fissions and fusions of chromosomes; in some cases, these can reduce or prevent gene flow. In the wild, prevention of gene flow between species is expected to, at least in part, maintain species integrity (Mallet, 2005). Within a crop (or between a crop and its wild relatives), these structural changes could potentially impede breeding attempts.

**FIGURE 1:**
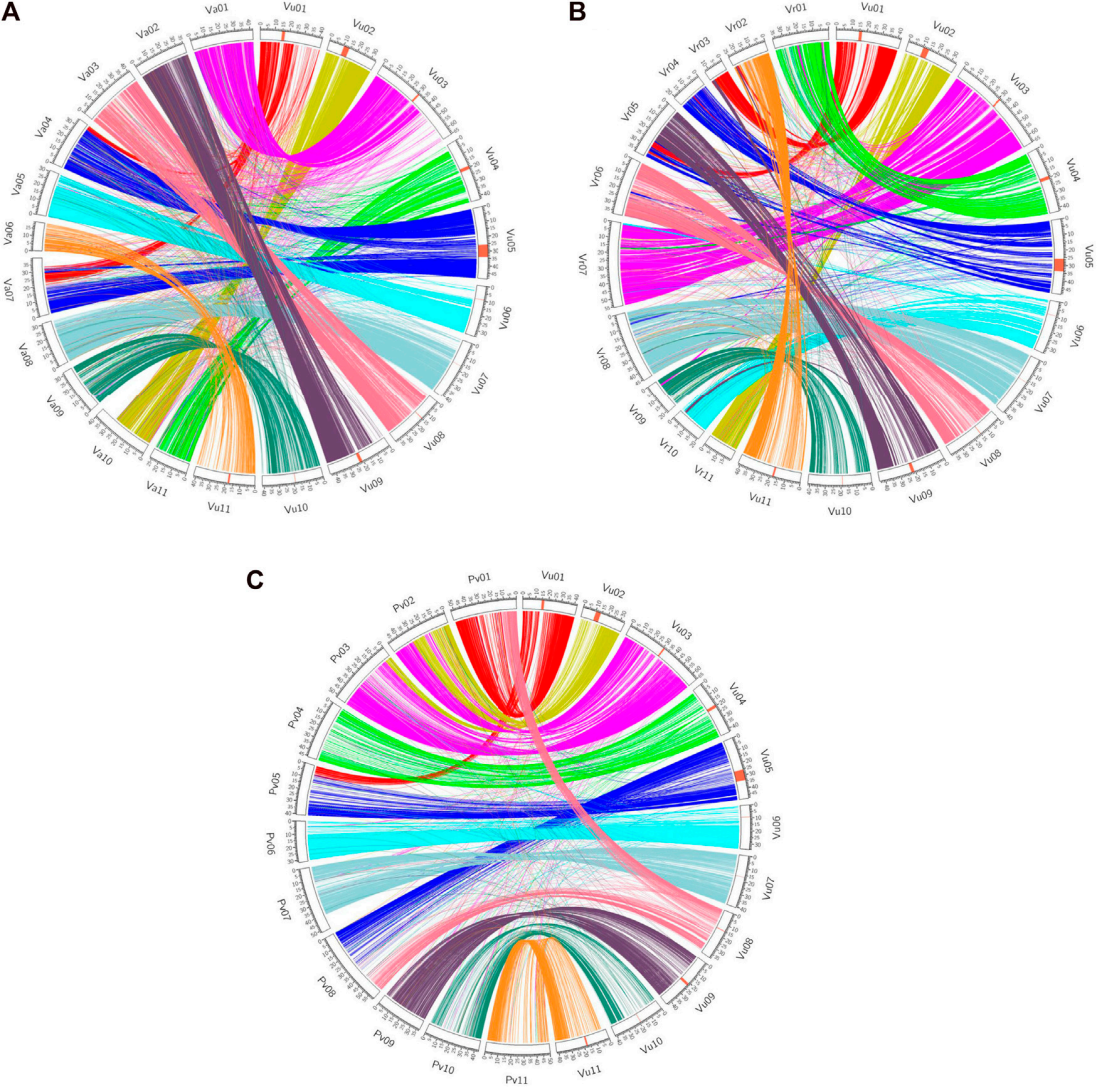
Synteny view between cowpea and adzuki bean **(A)**, mung bean **(B)** and common bean **(C)** [taken from Lonardi et al., 2019 (CC BY 4.0)].

Synteny analyses demonstrate how related species’ genomes ‘stack up’ against each other. Several scientists have used genetic markers to determine the rate of translocations, fissions and fusions over species divergences, e.g. in Solanaceous crops (Wu and Tanksley, 2010). However, these only provide a coarse scale determination of these parameters and would miss small-scale inversions and indels. For more fine-scaled analyses of both synteny, a whole genome sequencing approach must be pursued. Recent work, especially in legumes, have revealed how different species’ genomes compare in terms of gene order and chromosome similarity. Sequencing of the cowpea (*V. unguiculata*) genome and comparison to common bean was used to enumerate a chromosome numbering system in legumes. In this comparison six of the 11 chromosomes are largely syntenic, and five of the cowpea chromosomes are formed from parts of two common bean chromosomes (Lonardi et al., 2019). These were then compared to the genomes of adzuki and mungbean, thus revealing how the genomes have evolved during the last few million years. The cowpea genome analysis also revealed that a 4.2 Mb region of the genome was inverted in some accessions relative to others, highlighting how large structural variants are found both when comparing different species and within species (Lonardi et al., 2019; Figure 1). In a mapping population wherein the parents different in the orientation of the inversion there was an absence of recombination, a well-known effect of inversions (Kirkpatrick, 2010), and within this region was a gene putatively involved in resistance to the parasitic weed, *Striga* spp., although the functional significance remains to be explored (Lonardi et al., 2019).

Synteny analyses have been carried out for the minor legumes lablab (Njaci et al., 2023), moth bean (Yundaeng et al., 2019) and adzuki bean (Yang et al., 2015). This research reveals how the number of translocations, fissions and fusions increased with genetic distance. An in-depth analysis of synteny between lupin and multiple other legumes allowed for the determination of an ancestral legume karyotype as well as characterizing the lupin genome as being palaeotriploid (Hufnagel et al., 2020).

### 6.5 Cross-species and cross-genera marker transfer

Using a diverse panel of 98 wild and cultivated *Vigna* accessions from 13 species evaluated for agronomic traits for two seasons and genotyped with 92 cross-genera and cross-species SSRs, Kumari G. et al. (2022) detected three genetically distinct subpopulations and association of 13 SSRs with nine traits and seven markers associated with multiple traits. For example, VR022 for 100-seed weight and pod length; CEDG033 for days to flower and maturity; CEDG100 for 100-seed weight, plant height and terminal leaf length; CP1225 for chlorophyll content (CC) at 30 days, days to flower and maturity; and CEDG096A for CC30 and CC45 days, etc. CEDG100 co-localized in gene-encoding histone-lysine N-methytransferase *ATX5*, while VR22 co-located in gene-encoding *SHOOT GRAVITROPISM 5* in mungbean, thus suggesting these markers as potential genomic resource for marker-assisted genetic enhancement of mungbean and related *Vigna* species (Kumari G. et al., 2022).

## 7 QTL and genes associated with stress tolerance and agronomically beneficial traits

Drought and heat stress are adversely impacting agricultural production due to climate change. The food legume crops in general are more sensitive to abiotic stresses, while underutilized pulses serve as useful resource for allelic diversity associated with abiotic stress adaptations simply because of their inherent adaptation to inhospitable environments. Table 5 lists functionally characterized genes associated with domestication traits, pod/seed characteristics and abiotic stress adaptation in select minor grain legume crops. *Asr2*, *Dreb2B*, and *ERECTA* are key candidate genes that confer adaptation to drought. Assessing sequence variation of these genes between tepary bean and its wild relatives within *Phaseolus acutifolius* or *P. parvifolius* and comparing it with drought tolerance indices from climate data of geo-referenced tepary bean accessions reveals intermingling of cultivated and wild *P. acutifolius* alleles with var. *tenuifolius* and *P. parvifolius*. *Dreb2B* and *ERECTA* SNPs correlated with environmental drought indices, thus indicating that wild tepary beans are the source of novel alleles at genes for drought tolerance (Buitrago-Bitar et al., 2021). Associating sequence variation and GWAS data for growth sites at three widely divergent latitudes of rice bean landraces unlocked loci (*FUL*, *FT*, and *PRR3*) associated to the adaptation of rice bean from low to higher latitudes. Landraces pyramiding early flowering alleles for these loci were earliest to flower. Copy number variation for VumCYP78A6 regulate seed-yield traits, while an InDel in *TFL1* among landraces from mountainous region in South-Central China affect stem determinacy (Guan et al., 2022). Horse Gram is a drought hardy crop adapted to grow in marginal soils under receding moisture conditions. To date, a few genes (*MuWRKY3*, *MuNAC4, MuMYB96*, *MuHSP70*, *MuNAC4*) that confer drought tolerance in horse Gram were isolated and functionally characterized (Kiranmai et al., 2018; Masand and Yadav, 2016; Pandurangaiah et al., 2014). Ethylene-responsive factor (ERF) proteins are involved in plant growth and stress tolerance. Adzuki bean genome contains 47 *ERF* genes, of which 13 *ERF* genes were induced in response to saline-alkaline stress. Overexpression of *VaERF3* in transgenic *Arabidopsis* resulted in greater levels of proline accumulation and lower levels of malondialdehyde and reactive oxygen species in plants grown under saline-alkaline stress conditions (Li et al., 2020).

**TABLE 5:** Functionally characterized genes associated with photoperiod-induced flowering, seed nutritional quality and/or abiotic stress adaptation in adzuki bean, grass pea, horse Gram, lablab bean, rice bean, and tepary bean.

Gene	Function	References
Abiotic stress tolerance		
*MuMYB96*, *MuWRKY3*, *MuNAC4*	Simultaneous expression of *MuMYB96*, *MuWRKY3*, and *MuNAC4* in peanut improve stress tolerance and productivity of peanuts under drought stress
*Asr2*, *Dreb2B*, *ERECTA*	*Dreb2B* and *ERECTA* based SNPs correlated with the environmental drought indices, thereby wild tepary beans are the source of novel alleles for drought tolerance	Buitrago-Bitar et al. (2021)
*VaERF3*	Overexpression in *Arabidopsis* resulted in higher proline and lower malondialdehyde and ROS under saline-alkaline stress conditions	Li et al. (2020)
*MuWRKY3*	Overexpression in peanut improves tolerance	Kiranmai et al. (2018)
*MuHSP70*	*Arabidopsis* overexpressing *MuHSP70* maintains robust physiological traits including biomass and chlorophyll content under multiple stresses	Masand and Yadav (2016)
*MuNAC4*	Transgenic peanuts containing *MuNAC4* enhances drought adaptation	Pandurangaiah et al. (2014)
Photoperiod induced flowering		
*FD1*	Contains a single gene, *VaE1* in adzuki bean; greater *VaE1* expression in photoperiod sensitive line under LD than SD; lower *VaE1* expression in photoperiod insensitive line, regardless of day length	Imoto et al. (2022)
*LprPHYA3*	Modulates photoperiod-induced flowering in lablab bean	Krishna et al. (2022)
Seed quality		
*LpBADH2*	Substitution of tyrosine (fragrant) with phenyl alanine (non-fragrant) in protein coded by *LpBADH2* caused the switch from fragrance to non-fragrance in lablab bean	Basangouda et al. (2023)
*Ls*OCS	β-ODAP, synthesized from oxalyl-CoA and L-α,β-diaminopropionic acid; oxalyl CoA-synthetase isolated from grass pea; substituting *Ls*OCS with an oxalate oxidase or decarboxylase may reduce β-ODAP content	Goldsmith et al. (2022a)
*VaSDC1*	Low *VaSDC1* expression adzuki bean red-seed coat variety results in lower accumulation of anthocyanin, whereas its overexpression accumulates more anthocyanins in black seed coat adzuki bean variety	Chu et al. (2021)
Stem determinacy		
*TFL1*	An InDel in *TFL1* affect stem determinacy in rice bean	Guan et al. (2022)
*PvTFL1y*	Transition from G to A at the end of the third exon differentiated GNIB 21 (determinate) from GPKH 120 (indeterminate) in lablab bean	Kaldate et al. (2021)

Pulses with determinate growth habit are preferred for seed production because of early flowering and synchronous pod maturity, insensitivity to photoperiods, and mechanized harvesting or ease in manual harvesting. *PvTFL1y*, an common bean orthologue of *Arabidopsis TFL*, controls growth habit (Kwak et al., 2008), with many orthologs detected in other pulses, including lablab bean (Ramtekey et al., 2019). *PvTFL1y* amplifies *TFL* locus in indeterminate (GPKH 120) and determinate (GNIB 21) lablab bean germplasm. A splice site SNP in *TFL* locus confers determinate growth habit in GNIB 21. The transition from G to A at the end of the third exon differentiated GNIB21 from GPKH 120, which may modulate growth habit in other legumes through genome editing (Kaldate et al., 2021). Photoperiod sensitivity is a major factor in adzuki bean latitudinal adaptation. The QTL *FD1* has a large effect on photoperiod response to flowering. Imoto et al. (2022) delimited the *FD1* locus to a 17.1 kb sequence, containing a single gene, *VaE1*, an *E1* ortholog. A sequence comparison of photoperiod sensitive and insensitive adzuki bean lines revealed 29 indels and 178 SNPs upstream of *VaE1* in the *FD1* locus. *VaE1* expression was lower in photoperiod insensitive line irrespective of day length, indicating *VaE1* acts as a floral repressor by being upregulated under LD conditions. The inability to upregulate *VaE1* under LD is linked to its ability to flower under these conditions, which can be deployed in breeding adzuki beans adapted at higher latitudes (Imoto et al., 2022).

Variation in seed coat colour, in general, influence the synthesis and accumulation of phenolics and flavonoids, which exhibit significant antioxidant and radical-scavenging activities to prevent age-related noncommunicable diseases in humans (Dwivedi et al., 2022). *VaSDC1*, an R2R3-MYB TF, regulates seed coat color (black, red) in adzuki beans. *VaSDC1* expression activates the flavonoid metabolic pathways structural genes to substantially accumulate anthocyanins, while low *VaSDC1* expression results in a lower anthocyanin accumulation (Chu et al., 2021), suggesting *VaSDC1* could improve the nutritional quality of adzuki beans. Seeds containing high anthocyanins impart black/purple colour. Freshly harvested lablab bean pods have a unique fragrance that fetches a premium price. A sequence variant in *BADH* confers fragrance in soybean (Qian et al., 2022). A lablab bean homolog (*LpBADH2*) of soybean *GmBADH2* with a high degree of similarity (97%) contains one and three non-synonymous and synonymous SNPs, respectively. Substitution of the amino acid tyrosine (fragrant accessions) with phenylalanine (non-fragrant accessions) in the LpBADH2 protein suggests involvement in fragrance, which could be deployed in breeding programs to develop lablab bean cultivars with high fragrance (Basangouda et al., 2023).

Recently discovered putative candidate genes and QTL associated with agronomically useful traits are, respectively, summarized in Tables 6, 7. The examples include pod/seed, pod fiber and pod shattering traits in African yard long bean or moth bean (Yundaeng et al., 2019; Garcia-Oliveira et al., 2020; Watcharatpong et al., 2020); seed weight and seed size in rice bean (Verma A. et al., 2022); photoperiod-induced flowering in adzuki and lablab beans (Imoto et al., 2022; Krishna et al., 2022); relative water content, root traits (volume, weight, and length), chlorophyll content and biochemical pathways genes associated with abiotic stress adaptation in adzuki bean, Bambara groundnut, horse Gram, and moth bean (Katoch and Chahota et al., 2021; Tayade et al., 2022b; Choudhary et al., 2022; Meena et al., 2022; Odesola et al., 2023) or salt stress tolerance in grass pea (Alsamman et al., 2023); genes involved with unique nutritive values of moth bean seed (Suranjika et al., 2022); and anthocyanin contents in winged bean pod and seed (Chankaew et al., 2022).

**TABLE 6:** Candidate genes associated with abiotic stress adaptation, photoperiod-induced flowering, pod/seed size and quality traits in African yard long bean, adzuki bean, grass pea, horse Gram, lablab bean, moth bean, rice bean, and winged bean.

Trait group	Crop	Putative candidate gene	References
Abiotic stress	Adzuki bean	*VrLEA-2*, *VrLEA-40*, *VrLEA-47*, and *VrLEA-55* significantly upregulated under heat stress conditions in heat-tolerant genotypes	Singh et al. (2022)
		Significantly higher expression (*VaPIP2-1*, *VaPIP2-5*) in root or variable expression (*VaPIP1-1*, *VaPIP1-7*) in leaf linked with water uptake under drought stress or in osmoregulation to transport substrates rather than water to protect plants from drought	Tayade et al. (2022b)
	Grass pea	*LsbHLHD4, LsbHLHD5, LsbHLHR6, LsbHLHD8, LsbHLHR14, LsbHLHR68*, and *LsbHLHR86* confer tolerance to salt stress	Alsamman et al. (2023)
	Horse Gram	Increased mRNA transcripts of WRKY TF genes in drought stressed plants reduces the adverse effect of drought on plants	Kiranmai et al., 2016
	Lablab bean	Unfolded 17 genes associated with drought stress response, depending on the expression levels peaked after 6-, 8- or 10-day of dehydration	Yao et al. (2013)
	Moth bean	*VacoCAT1* associated with drought tolerance	Meena et al. (2022)
		Catalase, cyt P450 monooxygenase, HSP 90 and HSP 70, protein kinases, oxidoreductase, dehydration responsive protein, universal stress protein and ferridoxin NADH oxidoreductase genes upregulated in stressed sample	Tiwari et al. (2018)
Pod/seed size and quality	African yard long bean	*VuBGLU12* and *VuMYB26b* associated with pod fiber contents and pod shattering	Watcharatpong et al. (2020)
	Moth bean	Most of the 12,839 unigenes having differential expression in the late stages of seed development, possibly involved in unique nutritive values of seeds; annotated 74,082 unigenes as TFs	Suranjika et al. (2022)
	Rice bean	Auxin and cytokinin pathways possibly regulate seed weight; 51 genes encoding *SCF* ^*TIR1/AFB*^, *Aux/IAA*, *ARFs*, *E3* ubiquitin transferase enzyme, and *26S* proteasome with distinct expression dynamics between small and large-seeded lines	Verma A. et al. (2022)
	Winged bean	Transcriptome sequencing of leaves of two lines differing in condensed tannin (CT) revealed 5210 contigs involved in 229 different pathways; 1,235 contigs differentially expressed between high and low CT lines	Singh et al. (2017)

**TABLE 7:** Quantitative trait loci (QTL) associated with abiotic stress adaptation and domestication, morpho-phenological, pod/seed size and quality traits in adzuki bean, African yard long bean, Bambara groundnut, horse Gram, moth bean, and winged bean.

Trait group	Crop	QTL	References
Abiotic stress	Bambara groundnut	Eight QTL associated with stress tolerance index under drought stressed environments	Odesola et al. (2023)
	Horse Gram	7, 4, and 8 QTL associated with relative water content, root volume, and root length, respectively; genes on these marker sites involved in many biochemical pathways related to abiotic stresses	Choudhary et al. (2022)
		One QTL for malondialdehyde content on LG2, two QTL for root length on LG3 and LG9, one QTL each for proline and chlorophyll contents on LG4, and one QTL each for root dry weight and root fresh weight on LG 5	Katoch and Chahota (2021)
		Five QTL for four traits related to drought (days to temporary wilting, root length) and yield (seeds plant^-1^, days to maturity) on five LGs	Chahota et al. (2020)
Domestication related traits	African yard long bean	Between one and 11 QTL mapped on narrow genomic regions (LGs 3, 7, 8, 11); major QTL for sizes of seed, pod, stem and leaf on LG7	Kongjaimun et al. (2012a)
	Moth bean	Large effect QTLs with one or two minor QTLs control seed dormancy and pod shattering	Yundaeng et al. (2019)
	Rice bean	A few major QTL mapped as clusters on LGs 2, 4, and 7	Isemura et al. (2010)
Morpho-phenological traits	Adzuki bean	A major QTL on LG03 and two minor QTL on LG05 associated with flowering	Liu et al. (2016)
	Horse Gram	Four QTL for phenology (flowering, reproductive period, maturity) and seven QTL for morphological (plant height, primary and secondary branches) traits across environments	Katoch et al. (2022)
Pod/seed size and quality	African yard long bean	Major QTL for pod length (*qPoL3*) and seed breadth (*qSB4*), length (*qSL7.2*) and thickness (*qST9*) mapped; previously reported QTL for pod length (*qPoL8*) and 100-seed weight (*qSW8*) and for seeds pod^-1^ (*qSN9.2*) confirmed	Garcia-Oliveira et al., 2020
		One major and six minor QTL associated with pod length	Kongjaimun et al. (2012b)
	Winged bean	31 QTL linked with pod length, pod colour, pod anthocyanin content, and flower and seed colour; the major QTL for pod colour, anthocyanin content, and calyx colour, and for seed colour and flower wing colour located at the same position	Chankaew et al. (2022)

## 8 Biotechnology-led approaches to enhance productivity and stress tolerance

The underutilized grain legumes, unlike major pulses in the past, received less attention in crop improvement programs largely because of their low yields and restricted cultivation by resource-poor farmers in marginal lands. Of late it has been realized that such crops withstand abiotic stress adaptation much better, and grains are more nutritious including rich source of phytochemicals than traditional pulses. Increased emphasis on these crops in identifying novel sources of variation in gene pools, unfolding seed nutritional virtue as potential nutraceutical targets for functional foods, and unravelling the physiological and molecular basis of abiotic stress adaptation led to establishing high density genetic maps, mapping QTL, investigating putative and functionally characterized genes, and unlocking of marker-trait associations. These research advances set the stage for effective use of genomic-assisted breeding to select for enhanced productivity and abiotic stress adaptation in some of these underutilized pulses, as detailed herewith.

### 8.1 Biological constraints to introgression breeding

The introgression breeding method has been used to improve crops since domestication (Hernandez et al., 2020). It is an important method for the breeding of crops to improve their productivity and resilience under stresses caused by climate change (Gramazio et al., 2021). It involves the integration of genetic materials from one species to another, often from a wild relative to a domesticated crop (Quezada-Martinez et al., 2021). Wild crop relatives are sources of alleles for adaptation to unfavorable climatic conditions, and resistance to pathogens and pests (Hernandez et al., 2020; Gramazio et al., 2021).

Crops are domesticated through selection for desirable traits such as the selection of high-yielding cultivars under optimum conditions. However, this approach has led to a narrow genetic base that causes crop failure under climatic stress (Quezada-Martinez et al., 2021). As climate change ushers in unfavorable conditions, domesticated crops may not withstand the negative effect accompanied by these conditions. Introgression breeding can be used to transfer traits responsible for high productivity and stress tolerance from wild relatives to domesticated crop species for increased crop productivity and resilience. This method is essential for the improvement of underutilized crops by introgressing desirable traits from their wild relatives.

This method, however, is constrained by some biological factors such as the difficulty of hybridization, large genetic distance between the donor and recipient, low crossover frequency and distributions in the hybrid, and selection for desirable introgression while minimizing linkage drag can be tedious (Quezada-Martinez et al., 2021). Introgression involves the crossing of wild relatives with domesticated crop species and several backcrossing of the resulting hybrids with parents however if either the hybridization of the wild relative and domesticated crop is difficult or impossible introgression cannot take place as in the case of lima bean and its relatives’ common bean, and also with runner bean (*Phaseolus coccineus*) and tepary bean. The inability to hybridize could be caused by many factors, which can include large genetic distance between the wild relative and domesticated crop species. In cases where crosses were possible, the fertility of hybrids were mainly dependent on compatible chromosomal arrangement of the wild parent (Singh et al., 2018). Introgression can also be constrained by low level of gene flow in the hybrids, which leads to the non-expression of introgressed genes. Despite the challenges accompanied by introgression this method is effective in the transfer of desired traits from wild relatives to domesticated underutilized crops.

### 8.2 Reducing toxin (neurotoxin) in grass pea

β-ODAP content varied widely (0.02%–2.59%) among *Lathyrus* germplasm, with greater content in stressed environments. A few wild relatives such as *L. cicero*, *L. amphicarpus* and *L. ochrus* have zero or low β-ODAP (≤0.01%). They may be utilized for the development of toxin free *Lathyrus* cultivars. Crossbreeding has resulted in the release of several high yielding grass pea cultivars with low β-ODAP content in many countries in Africa, Asia, Australia, and America. Impressive progress has been achieved towards applying biotechnological resources, including transgene technology, in the genetic enhancement of grass peas (Das et al., 2021).

Lack of rapid screening technique for identification of low β-ODAP germplasm or segregants in breeding populations is the major impediment to grass pea breeding. A recently developed high throughput plate assay based on spectrophotometric method allows quantification of total β-ODAP in a large number of samples, but its low sensitivity and inability to differentiate α- and β-L-ODAP limits its usefulness. Use of stable isotope as internal standard with a novel liquid chromatography mass spectrometry (LCMS)-based method for β-L-ODAP quantification facilitates accurate identification and characterization of grass pea lines with a very low ODAP content (Emmrich et al., 2019). An improved HILIC-MS/MS method without sample derivatization determines both toxic (β-ODAP) and nontoxic (α-ODAP) isomers in grass pea. It uses a hydrophilic interaction chromatography (HILIC) column and an isocratic gradient of eluents to determine both α- and β-ODAP contents. β-ODAP content in a validation study involving 107 geographically diverse grass pea accessions ranged between 0.45 and 6.04 mg g^-1^ dry seeds, differentiated contrasting accessions, and showed moderate correlation (0.65) between α- and β-ODAP contents, reinforces independent quantification of both ODAP isomers (Bento-Silva et al., 2019).

β-ODAP synthase (BOS), a recently discovered enzyme of the benzylalcohol O-acetyltransferase, anthocyanin O-hydroxycinnamoyltransferase, anthranilate N-hydroxycinnamoyl/benzoyltransferase, deacetylvindoline 4-O-acetyltransferase superfamily of acyltransferases, provides catalytic activity linked with β-ODAP formation. It is structurally similar to hydroxycinnamoyl transferase. BOS expression in the presence of its substrates causes β-ODAP production *in vivo*, which may pave the way to engineer β-ODAP–free grass pea cultivars (Goldsmith et al., 2022a). Oxalic acid, a small metabolite with several metabolic pathways to control oxalate levels by enzymatic degradation, protects plants from herbivores damage. *LsOCS*, grass pea oxalyl CoA-synthetase gene, encodes a monomeric protein of 56 kDa, having catalytic efficiency with oxalate similar to that of *Arabidopsis thaliana* (*AtAAE3*) and *Medicago truncatula* (*MtAAE3*) homologs. Substituting *LsOCS* with oxalate oxidase or decarboxylase could reduce the level of β-ODAP in grass peas. Inactivating *LsOCS* in grass pea using genetic engineering has potential to reduce the biosynthesis of β-ODAP, however, it may increase grass pea susceptibility to pathogens such as *Sclerotinia sclerotiorum*. Thus, replacing *LsOCS* with an exogenous oxalate-oxidase or decarboxylase could enable plants to regulate cellular oxalate levels while reducing the levels of β-ODAP in grass pea (Goldsmith et al., 2022b). Germplasm with low β-ODAP may show reduced stress tolerance due to reduced relative water content and perturbed abscisic acid levels (Verma S. K. et al., 2022).

### 8.3 Enhancing bruchid resistance to minimize losses during storage

Beetles (also known as weevil, Callosobruchus maculatus) cause substantial damage to pulse grains during storage. Early research shows that *Arcelin* (Arl), an insecticidal gene from lablab bean, is homologous to Arl-3 and Arl-4 alleles from Phaseolus spp, with about 70% amino acid similarity. The artificial diet containing *Arl* (0.2% w/w arcelin-incorporated artificial seeds) retarded growth of cowpea weevil, which may be deployed to incorporate bruchid resistance through transgenesis in pulses (Sundaram et al., 2012). Exceptionally high *Arl* gene expression in wild sward bean (*Canavalia virosa*) suggests it may be used to develop weevil resistance in other cultivated pulses (Sakthivelkumar et al., 2014). An insecticidal activity of *Arl* gene, isolated from a wild accession of lima bean (*Phaseolus lunatus*), drastically reduces adult emergence and seed damage, thereby demonstrating the effectiveness against the bruchid beetle (Hilda et al., 2022).

The single dominant gene *Rcc* confers beetle resistance in moth bean. One major (qVacBrc2.1) and one modifying (qVacBrc5.1) QTL residing in *Rcc* control resistance. qVacBrc2.1, mapped on LG2 between SSRs CEDG261 and DMB-SSR160, accounted for 50%–64% variation for resistant traits. *qVacBrc2.1* is the same as QTL *Brc2.1*, which confers beetle resistance in wild adzuki bean (Somta et al., 2018). Fine mapping revealed two novel markers associated with qVacBrc2.1 constituted two linked QTL, qVacBrc2.1-A and qVacBrc2.1-B, and two polygalacturonase-inhibiting protein genes, VacPGIP1 and VacPGIP2 as candidate genes for beetle resistance in TN67. The alignment of *VacPGIP1* coding sequences between TN67 (resistant) and ICMP0056 (susceptible) accessions revealed eight SNPs, three of which altered the amino-acid sequence of the predicted domains of polygalacturonase inhibitors in ICPMO056 (Gamage et al., 2022).

### 8.4 Rice bean introgression to enhance stress tolerance and productivity of black gram

*Mungbean yellow mosaic India virus* (MYMIV) causes significant yield losses in pulses including black gram (*Vigna mungo*) and green gram (*Vigna radiata*). High level of resistance to MYMIV was reported in a black gram variety Mash114. A large effect QTL, qMYMIV6.1.1 spanning 3.4 Mb on chromosome 6, identified as an inter-specific introgression from rice bean, accounted for 70% of total phenotypic variation. KASP markers closely associated with MYMIV delineated 500 kb genomic region linked with MYMIV, which can be deployed for marker-assisted transfer of introgressed region into improved genetic backgrounds of *Vigna* species (Dhaliwal et al., 2022). Advanced lines originating from a rice bean and black gram cross showed wide variation in seed yield, ranged from −35.48 to +50.31% over control (Mash338), and were found resistant to MYMIV, *Cercospora* leaf spot and bacterial leaf spot. The superior yield performance and disease resistance traits were introduced from the rice bean genotype KUG114, and offspring with yield superiority of ∼39% over ‘Mash338’ has been released as “Mash114” for cultivation in Punjab (Singh et al., 2013).

### 8.5 Tepary bean introgression to enhance stress tolerance and productivity of common beans

The genus *Phaseolus* contains many species, including tepary bean, a valuable genetic resource for abiotic stress tolerance and productivity genes. Wild tepary bean accession W6 15,578 is a potential donor for cold tolerance. A three-year field assessment of an interspecific backcross population derived from the cross W6 15,578 × NY5-161 (common bean) led to identifying lines tolerant to sub-zero temperature at seedling stage. Their subsequent evaluation revealed that few outyielded their common bean parent under cold stress and drought, thereby suggesting that introgression of a proportion of tepary bean genome into common bean is a promising strategy to enhance abiotic stress adaptation in the latter (Souter et al., 2017). However, obtaining hybrids between tepary and common bean is problematic (i.e., hybrid sterility) and therefore pollination technique, growth conditions and embryo rescue methods are used for successful introgression of tepary genes into common bean (Andradf-Aguilar and Jackson, 1988; Scott and Michaels, 1990; Pratt and Gordon, 1994).

Barrera et al. (2022) recycled novel interspecific derived lines obtained from common bean × tepary bean and hybridized these to *Phaseolus parvifolius* to increase male gametic diversity to facilitate interspecific crossing. Introgression of such lines enhanced the success of common bean and tepary bean hybridization without the use of embryo rescue technique and resulted in a 12-fold more hybrid plants than crossing directly between common beans and tepary beans. Such lines contain large introgression of genomic regions from *P. parvifolius*, thus providing means to efficiently exploit tepary gene pool for enhancing abiotic stress adaptation in common bean without the need for embryo rescue (Barrera et al., 2022).

A multi-environment evaluation of interspecific congruity backcross lines, obtained from common bean and tepary bean cross, resulted in a few lines that produced high yield under extreme weather conditions of coastal Colombia, thereby suggesting that it is feasible to combine drought and heat stress tolerance, as evidenced in line 68, with high Fe mineral biofortification (Burbano-Erazo et al., 2021). A few elite lines with large seeds and erect plant architecture, abiotic stress adaptation, and resistance to bacterial blight and weevil may be recycled to enhance productivity and stress tolerance traits in common bean (Porch et al., 2013; 2022).

### 8.6 Enhancing abiotic stress adaptation using horse gram genes

*Arabidopsis* transgenics overexpressing *MuHSP70*, a gene cloned from horse Gram, exhibit multiple abiotic stress tolerances, which result in greater shoot biomass, root length, relative water content, and chlorophyll content during multiple stresses (Masand and Yadav, 2016). Transgenic peanuts containing *MuNAC4*, another gene from horse Gram, exhibited significantly enhanced drought tolerance due to increased later roots and greenish growth by reducing damage to membrane structures and enhancing osmotic adjustment and antioxidant enzyme regulation under stress (Pandurangaiah et al., 2014).

*MuWRKY3* expression alone or simultaneous co-expression with other stress-responsive regulatory TFs genes (*MuNAC4*, *MuMYB96*), all cloned from horse Gram, improved drought tolerance in peanuts (Kiranmai et al., 2018). Transgenic plants showed increased growth of lateral roots, chlorophyll content, stay-green, and maintained higher relative water content compared to WT. Expression analysis of transgenes and their downstream regulatory genes revealed two-to four-fold increase in transcript levels under drought stress in multigene transgenic peanut plants over WT. Thus, multiple genes transfer with simultaneous expression is a promising option to improve stress tolerance and productivity in peanuts and possibly in other pulses in drought stressed environments.

## 9 Research gaps in the study and genetic improvement of underutilized pulses

The pulses we have focused on have lagged in the development and use of genetic and genomic resources in applied breeding compared to more mainstream legume crops. The priority areas of research over the past three decades have been to develop and use these types of resources for uplifting the productivity of major food legume crops (Boukar et al., 2016; Assefa et al., 2019; Bohra et al., 2020; Pandey et al., 2020; Arriagada et al., 2022; Peterein et al., 2022). The declining production and nutritional quality of staple food crops produced today due to climate change and variability effects (Dwivedi et al., 2013; Myers et al., 2017; Soares et al., 2019) has forced policymakers to look for alternative crops to enhance food and nutritional security (Myers et al., 2017; Searchinger et al., 2019). Among the underutilized pulses discussed here we see crops such as horse gram and lablab bean that are better adapted to marginal soils and nutritionally dense grains. They are, however, typically low yielding. Hence, enhancing the productivity of such legume crops without diluting their stress tolerance or nutritional quality demands a paradigm shift in breeding by infusing knowledge-led genetic improvement.

Underutilized pulses included in this review have different levels of resources available (both genetic and genomic) (Table 8). These crops, except for adzuki bean, Bambara groundnut, grass pea and lima bean, are underrepresented in genebanks (Table 2). Small and marginal farmers are the primary cultivators, though on a limited acreage, and often using their own-saved seed source, passed perpetually in the family. Efforts should be directed to collect, conserve, characterize, preserve, and document on-farm diversity and the associated knowledge. Diversity panels and reduced subsets in the form of core collections are ideal genetic resources to dissect structure and diversity and for conducting GWAS. Advanced mapping populations (RILs) including those in the form of NAM or MAGIC design, and the development of DNA markers are required to construct high density genetic linkage maps to ultimately clone gene(s) of interest. Such resources are limited in underutilized pulses; for example, only five of the 13 crops included in this review have SNP markers available, ranging from a few hundred to a few thousand, whereas for the other crops very limited numbers of other categories of markers are available. Six crops have high density genetic maps available, while the genomes of seven crops with good contiguity and coverage have been generated. Thus, marker development and genome sequencing should be priority of research in several of the crops we have discussed.

**TABLE 8:** Advances in levels of genetic and genomic research and research gaps amongst 13 underutilized pulses.

Levels of research on genetic resources		Levels of research on genomic resources			Research gap
Core collection	Diversity assessment^a^	DNA marker^b^	High density genetic map	Genomes and pangenomes	
African yam bean (*Sphenostylis stenocarpa*)					
	5	5,511^†^			Conservation of landraces and on-farm diversity
					Core collection
					Genetic map and genome sequencing
Adzuki bean (*Vigna angularis*)					
√	6	221^‡^	√ (Han et al., 2005)	√	Conservation of landraces and on-farm diversity
		187^§^			SNPs markers
		94^Ψ^			Marker-based diversity assessment
Bambara groundnut (*Vigna subterranea*)					
√	11	17,481^†^	√ (Gao et al., 2023)	√	Conservation of landraces and on-farm diversity
					Multi-season phenotype-based diversity assessment
Grass pea (*Lathyrus sativus*)					
	5	135^‡^	√(Hao et al. (2022)	√	Core collection
		GBS-derived 3536^†^ (Hao et al., 2022)			SNPs markers
Horse gram (*Macrotyloma uniflorum*)					
	5	300^‡^		√	Core collection
					Multi-season phenotype-based diversity assessment
					SNP markers
Kersting’s groundnut (*Macrotyloma geocarpum*)					
	2	1379^†^			Conservation of landraces and on-farm diversity
					Core collection
					Marker- and multi-season phenotype-based diversity assessment
					Genetic map and genome sequencing
Lablab bean (*Lablab purpureus*)					
√	4	12,780^†^		√	Genetic map
					Marker- and multi-season phenotype-based diversity assessment
Lima bean (*Phaseolus lunatus*)					
√	6	10,497^†^ (Garcia et al., 2021)	√ (Garcia et al., 2021)	√	SNP markers
		103^‡^			Marker- and multi-season phenotype-based diversity assessment
Moth bean (*V. aconitifolia*)					
√	1	172^‡^			Conservation of landraces and on-farm diversity
					Marker- and multi-season phenotype-based diversity assessment
					SNP markers
					Genome sequencing
Rice bean (*V. umbellata*)					
	3	41^‡^			Core collection
					Conservation of landraces and on-farm diversity
					Marker- and phenotype-based diversity assessment
					Genetic map and genome sequencing
Tepary bean (*P. acutifolius*)					
	3	768^†^	√(Gujaria-Verma et al., 2016)	√	Core collection
		13^‡^			Conservation of landraces and on-farm diversity
					Marker- and multi-season phenotype-based diversity assessment
Winged bean (*Psophocarpus tetragonolobus*)					
	3	1384^†^; 27^‡^	√ (Chankaew et al., 2022)	√	Core collection
					Conservation of landraces and on-farm diversity
					Population structure and diversity
Yard long bean (*V. unguiculata* ssp. *unguiculata* cv.-*gr. Sesquipedalis*)					
	1	26^§§^			Core collection
					Conservation of landraces and on-farm diversity
					SNP markers
					Population structure and diversity
					Genetic map and genome sequencing

^a^ Includes both morphological (at least two seasons‘ data) and marker-based assessment reported in Table 4.

^b^ DNA markers used in diversity assessment reported in Table 5; ^†^, SNPs; ^‡^, SSRs; ^§^, AFLPs; ^ᴪ^, RFLPs; ^§§^, Indels; √, Available.

The levels of research also vary among underutilized pulses. While some crops such as African Yam bean, adzuki bean, Bambara groundnut, grass pea, horse Gram, lablab bean, and lima bean have had their germplasm to some extent characterized for genetic diversity (Table 8), such reports on other underutilized pulses are scant. QTL mapping has led to the discovery of genomic regions and candidate genes associated with stress tolerance and morpho-agronomic traits (Tables 6, 7). However, only a few genes have been functionally characterized (Table 5). For example, overexpression of single horse Gram genes (*MuWRKY3*, *MuHSP70*, *MuNAC4*) or simultaneous expression of *MuMYB96*, *MuWRKY3*, and *MuNAC4* provided multiple abiotic stress adaptation in Arabidopsis or groundnut and overexpression of a lablab R2R3-MYB gene in *Arabidopsis* increased drought and salt tolerance (Yao et al., 2016). Clearly, the adoption of these resources in breeding is currently very low, while such resources are routinely deployed in genetic improvement of major legume crops (Peterein et al., 2022; Salgotra and Stewart Jr., 2022).

## 10 Concluding remarks and future perspectives

Underutilized pulses are often adapted to harsh environments and their seeds are nutritionally packed with protein, fiber, minerals, vitamins, and phytochemicals. Although inherently low yielding, they produce where crops such as maize, rice, and wheat fail. These minor legumes are still underutilized because they possess some phenotypically undesired traits, which can be removed or suppressed while improving desirable traits through breeding methods.

One of the first steps to breeding for improved cultivars of underutilized legumes should be to track viable materials, use consistent labelling system and use a universal descriptor to phenotype germplasm. Hence, precision-led characterization and optimum conservation of accessions are required to provide adequate information for selection, which is the basis for crop improvement. Furthermore, reference genome, pangenomics, and population-level sequencing are important in investigating the crop genome of underutilized legumes to know where alleles responsible for adaptive traits lie. This knowledge may help accelerate the breeding of underutilized pulses to improve their productivity under harsh environmental conditions.

The degree of research investment varied, with some crops researched more than other underutilized grain legumes. Presently, a few functionally characterized genes impacting stem determinacy, photoperiod induced flowering, stress tolerance, and nutritional quality as well numerous QTL and putative candidate genes associated with domestication related traits, pod and seed characteristics, stress tolerance, and seed quality were uncovered, they may be deployed, after validation, in genomic-aided breeding. Such genomic resources will also be helpful in detecting introgression of these genes from wild relatives to cultivated type and vice versa, as well as for *de novo* domestication of crops.

Future research on underutilized pulses should focus on on-farm diversity and preservation of indigenous knowledge associated with cultivation and use of underutilized legumes, standard crop ontology and regeneration protocol to catalogue and preserve diversity, machine learning and algorithms to obtain robust phenomics data on minor legume germplasm characterization, whole genome resequencing involving diverse germplasm, and genetic stocks with specific attributes.
